# Structure-Based Design of Novel Tetrahydro-Beta-Carboline Derivatives with a Hydrophilic Side Chain as Potential Phosphodiesterase Inhibitors

**DOI:** 10.3390/scipharm84030428

**Published:** 2015-09-26

**Authors:** Ahmed K. Elhady, Sara C. Sigler, Nazih Noureldin, Joshua C. Canzoneri, Nermin S. Ahmed, Gary A. Piazza, Ashraf H. Abadi

**Affiliations:** 1 Department of Pharmaceutical Chemistry, Faculty of Pharmacy and Biotechnology, German University in Cairo, Cairo 11835, Egypt; ahmed.elhady@guc.edu.eg (A.K.E.); nazih.noureldin@guc.edu.eg (N.N.); nermin.salah@guc.edu.eg (N.S.A.); 2 Department of Oncologic Sciences and Pharmacology, Drug Discovery Research Center, Mitchell Cancer Institute, University of South Alabama, Mobile, AL 36608, USA; sara_sigler@hotmail.com (S.C.S.); jcanzoneri@adtpharma.com (J.C.C.); gpiazza@health.southalabama.edu (G.A.P.)

**Keywords:** Terahydro-beta-carboline, phosphodiesterase 5 inhibitors, stereochemistry, structure-based design

## Abstract

Tadalafil is a clinically approved phosphodiesterase-5 inhibitor for the treatment of erectile dysfunction and pulmonary arterial hypertension. It contains two chiral carbons, and the marketed isomer is the *6R*, *12aR* isomer with a methyl substituent on the terminal nitrogen of the piperazinedione ring. In this report, tadalafil analogues with an extended hydrophilic side chain on the piperazine nitrogen were designed to interact with particular hydrophilic residues in the binding pocket. This leads to analogues with moderate inhibitory activity on phosphodiesterase-5, even for isomers in which chiral carbons are of the *S* configuration.

## 1. Introduction

**Figure 1:**
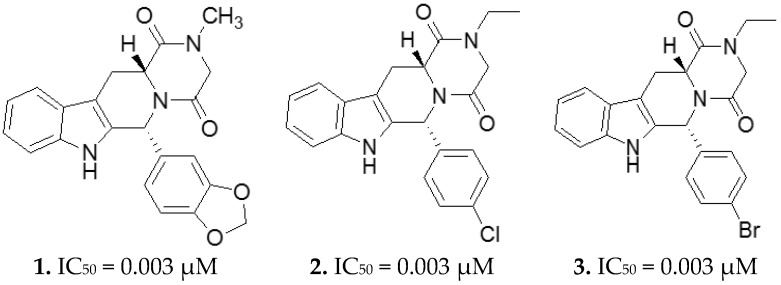
Chemical structures and IC_50_s versus phosphodiesterase-5 (PDE5) of Tadalafil (**1**) and other previously reported tadalfil analogues (**2**) and (**3**).

Phosphodiesterase-5 (PDE5) enzyme is a member of the class I phosphodiesterase superfamily [1,2]. It is considered a metallophosphohydrolase that is specific for the breakdown and degradation of cyclic guanosine monophosphate (cGMP) [3]. PDE5 is widely distributed in various tissues such as vascular smooth muscles, the brain, platelets, spleen, and kidney. Accordingly, PDE5 inhibitors have gained great attention as potential therapeutic agents for many indications, the most important being their use in the treatment of male erectile dysfunction (MED), where inhibition of PDE5 in the corpus cavernosum will prevent the breakdown of cGMP, lowering Ca^2+^ levels, which will relax the penile smooth muscles and thus maintain the engorgement of the penis under sexual stimulation [4]. Moreover, PDE5 inhibitors have been recently approved for the treatment of idiopathic pulmonary hypertension [5], where studies have shown their ability to decrease pulmonary vascular resistance, pulmonary arterial pressure, and right ventricular hypertrophy, leading to improvement in the cardiac indices that occur in response to the elevation of cGMP [6,7,8,9]. Tadalafil **1** is one of the most potent, clinically approved PDE5 inhibitors on the market. Several research groups have worked on the design and synthesis of many tadalafil analogues, using different aryl/heteroaryl substituents at C5/C6, and different alkyl/aryl substitutions at N2; in addition, all possible diastereomers were manipulated (*R*,*R*), (*R*,*S*), (*S*,*R*), (*S*,*S*). The most potent of them were compounds **2** and **3** with an IC_50_ of 3 nM, which is equipotent to that of tadalafil, Figure 1. The high potency for these three compounds can be explained through the reported crucial interactions with the residues in the PDE5 active site. These interactions include a hydrogen bond to **Gln817**, and hydrophobic interactions with the residues lining the P-clamp; the most important of which is **Phe820**, **Val782**, **Ile813**, and **Leu804** [10]. Further in silico studies suggested two more essential interactions between these three potent compounds with critical amino acids lining the PDE5 pocket. The first is the interaction between the terminal alkyl side chain on the N of the terminal ring with **Ile665**. The second is the hydrophobic interaction of the pendant aryl with **Met816** [11]. However, the interaction with **Ile665** has not been validated experimentally. The reason is the fact that this part of the enzyme (H-loop) containing this residue could not be co-crystallized due to its motility and as a result, it is absent in the PDE5 co-complex with tadalafil (PDB: 1UDU and 1XOZ). This work was done to confirm the hypothesized role of the **Ile665** interaction through structure-based design, where replacing the N-alkyl side chain of piperazinedione with one having a terminal polar group in the *R*,*R* diastereomer (the most active conformer) was expected to interfere with the hydrophobic interaction with **Ile665**, thus decreasing the potency of PDE5 inhibition.

Most studies reported that analogues with the C6 *S* absolute configuration yield essentially inactive analogues [11,12,13,14]. It has been recently shown by an ensemble docking study designed to explore the H pocket of PDE5 that the (*6S*, *12aS*) diastereomer only kept the interaction with **Phe820**, and lost all the other essential interactions, including those with **Gln817**, **Val782**, **Ile665**, and **Met816**. This study further indicated the close vicinity of **Asn661**, **Asn662** & **Ser663** residues to the **Ile665** residue, which suggests replacing the *N*-alkyl group with polar acceptor atom-containing groups or with halogen atoms to interact with these hydrophilic residues (**Asn661**, **Asn662**, & **Ser663**) having a polar, uncharged side chain, leading to novel PDE5 inhibitors with improved potency and selectivity. The study also revealed the close proximity of the hydrophilic residues (**Asn661**, **Asn662**, & **Ser663**) to the *S*,*S* isomer more than the *R*,*R* enantiomer [15].

**Figure 2:**
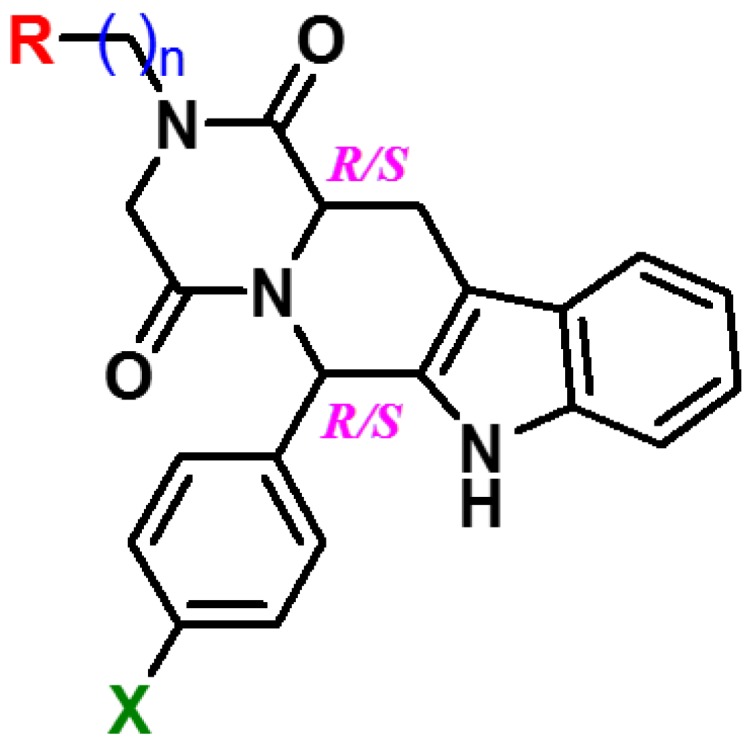
Novel PDE5 inhibitors with a hydrophilic side chain. n = 2, R = OH, NH_2_; n = 4, X = Cl, Br.

To experimentally test both hypotheses, 32 novel compounds were prepared with the following structural modifications as shown in Figure 2.

## 2. Materials and methods

### 2.1. Chemistry

All chemicals were obtained from Sigma-Aldrich & Alfa Aesar and were used without further purification. Solvents were obtained from commercial suppliers and were used without further purification. Melting points were determined on a Mettler FP1, melting point apparatus (Mettler Toledo, OH, USA) and are uncorrected. FT-IR spectra were recorded on the Nicolet Avatar 380 spectrometer. The NMR spectra were measured in DMSO-d6 or Acetone-d6 at RT using a Bruker DRX-500 spectrometer (Billerica, MA, USA). δ is given in ppm relative to tetramethylsilane as an internal reference. Silica gel column chromatography was carried out using silica gel 230–400 mesh, 60 Å (Sigma-Aldrich, St. Louis, MO, USA). Mass spectrometric analysis (HPLC-ESI-MS) was performed on a TSQ quantum (Thermo Electron Corporation, Waltham, Massachusetts) instrument equipped with an ESI source and a triple quadrupole mass detector (Thermo Finnigan, San Jose, CA, USA). The MS detection was carried out at a spray voltage of 4.2 kV, a nitrogen sheath gas pressure of 4.0 × 10^5^ Pa, an auxiliary gas pressure of 1.0 × 10^5^ Pa, a capillary temperature of 400 °C, and capillary voltage of 35 V. The progress of the reaction was monitored by Thin Layer Chromatography (TLC) using fluorescent pre-coated silica gel plates and detection of the components was made by short ultraviolet light at λ = 254 nm, and methylene chloride–methanol was used as an eluting system, with different ratios for each reaction.

#### 2.1.1. General Procedure for the Preparation of d- & l-Tryptophan Methyl Ester **4a**, **4b**

A 250-mL round-bottom flask containing methanol (140 mL) was cooled in an ice water bath, then solid d- or l-tryptophan (20 g, 97.93 mmol) was added in one portion. The solution was stirred for 5 min, then acetyl chloride (25 mL) was added dropwise using a dropping funnel over a period of 15 min, the solution was then heated to reflux for 5 h, allowed to cool to room temperature, and the solvent was removed under reduced pressure to give tryptophan methyl ester hydrochloride. The free base was obtained by adding diluted NH_4_OH and extraction was achieved with CH_2_Cl_2_ (3 × 50 mL). The organic layer was dried over anhydrous MgSO_4_ and evaporated under reduced pressure to give a yellow oil which solidifies upon cooling. It was used without further purification.

#### 2.1.2. General Procedure for the Preparation of Compounds **5a**–**5h**

10 g, 45.87 mmol of **4a** or **4b** was added to an aromatic aldehyde, namely 4-chlorobenzaldehyde and 4-bromobenzaldehyde (50.46 mmol), and dissolved in CH_2_Cl_2_ (50 mL). The solution was cooled to 0 °C in an ice bath. To this solution, TFA (8 mL) was added dropwise, and the mixture was stirred at room temperature for 4 days under N_2_ atmosphere. The reaction mixture was then basified with diluted NH_4_OH solution and extracted with CH_2_Cl_2_ (3 × 50 mL). The organic layer was washed with water, brine, dried over anhydrous MgSO_4_, filtered, and evaporated under reduced pressure. The residue was purified and the isomers were separated by column chromatography on silica gel using a CH_2_Cl_2_/CH_3_OH mixture (99:1) as an eluent, to give first the appropriate *cis* isomer followed by the *trans* one.

*(1R,3R)-1-(4-Chlorophenyl)-2,3,4,9-tetrahydro-1H-β-carboline -3-carboxylic acid methyl ester* (**5a**). Yellow solid. Yield: 30%. mp 220–223 °C. TLC, R_f_: 0.72. IR: 3375.1 (NH), 1737.3 (CO). ^1^H-NMR (DMSO-*d*_6_) δ: 2.91–2.82 (m, 1H, C*H_a_*H_b_), 3.09–3.02 (m, 1H, CH_a_*H_b_*), 3.72 (s, 3H, OC*H_3_*), 3.91–3.86 (dd, *J* = 10.93, 4.16 Hz, 1H, C*H*COOCH_3_), 5.23 (s, 1H, C*H*Ph), 7.05–6.94 (m, 2H, Ar-*H*), 7.24–7.21 (m, 1H, Ar-*H*), 7.34–7.31 (m, 2H, Ar-*H*), 7.46–7.43 (d, *J* = 7.49 Hz, 1*H*, Ar-*H*), 7.56–7.53 (m, 2H, Ar-*H*), 10.3 (s, 1H, N*H*). ^13^C-NMR (DMSO-*d*_6_) δ: 172.52, 141.31, 136.29, 134.66, 130.96, 130.66, 126.34, 120.70, 120.60, 118.27, 117.37, 110.99, 106.91, 56.98 (C1), 56.06 (C3), 51.50, 25.16.ESI-MS: m/z 343 (M^+^ + 2), m/z 341 (M^+^).

*(1S,3R)-1-(4-Chlorophenyl)-2,3,4,9-tetrahydro-1H-β-carboline-3-carboxylic acid methyl ester* (***5b***). Yellow solid. Yield: 15%. mp 189–191 °C. TLC, R_f_: 0.43. IR: 3334.5 (NH), 1709.6 (CO). ^1^H-NMR (DMSO-*d*_6_) δ: 2.95–2.87 (dd, *J* = 15.21, 7.32 Hz, 1H, C*H_a_*H_b_), 3.10–3.04 (dd, *J* = 15.17, 5.14 Hz, 1H, CH_a_*H_b_*), 3.63 (s, 3H, OC*H_3_*), 3.8–3.75 (m, 1H, C*H*COOCH_3_), 5.32 (s, 1H, C*H*Ph), 7.06–6.97 (m, 2H, Ar-*H*), 7.24 (t, *J* = 7.37 Hz, 3H, Ar-*H*), 7.53–7.44 (m, 3H, Ar-*H*), 10.61 (s, 1H, N*H*). ^13^C-NMR (DMSO-*d*_6_): δ 173.47, 142.26, 136.05, 133.62, 130.80, 130.26, 126.35, 120.72, 120.14, 118.22, 118.43, 110.88, 106.56, 53.29 (C1), 51.71 (C3), 51.37, 24.52.ESI-MS: m/z 343 (M^+^ + 20), m/z 341(M^+^).

*(1S,3S)-1-(4-Chlorophenyl)-2,3,4,9-tetrahydro-1H-β-carboline-3-carboxylic acid methyl ester* (**5c**). Yellow Solid. Yield: 37.5%. mp 221–224 °C. TLC, R_f_: 0.72. IR: 3382.7 (NH), 1726.2 (CO). ^1^H-NMR (DMSO-*d*_6_) δ: 2.90–2.82 (m, 1H, C*H_a_*H_b_), 3.08–3.04 (m, 1H, CH_a_*H_b_*), 3.70 (s, 3H, OC*H_3_*), 3.89–3.85 (m, 1H, C*H*COOCH_3_), 5.20 (s, 1H, C*H*Ph), 7.03–6.95 (m, 2H, Ar-*H*), 7.32–7.21 (m, 3H, Ar-*H*), 7.54–7.42 (m, 3H, Ar-*H*), 10.32 (s, 1H, N*H*). ^13^C-NMR (DMSO-*d*_6_): δ 172.63, 141.12, 136.45, 134.64, 131.21, 130.98, 126.45, 120.95, 120.91, 118.54, 117.65, 111.24, 107.03, 57.17 (C1), 56.15 (C3), 51.86, 25.21.ESI-MS: m/z 343 (M^+^ + 2), m/z 341 (M^+^).

*(1R,3S)-1-(4-Chlorophenyl)-2,3,4,9-tetrahydro-1H-β-carboline-3-carboxylic acid methyl ester* (***5d***). Yellow solid. Yield: 18.75%. mp 190–193 °C. TLC, R_f_: 0.43. IR: 3333.3 (NH), 1709.4 (CO). ^1^H-NMR (DMSO-*d*_6_) δ: 2.97–2.90 (dd, *J* = 15.18, 7.38 Hz, 1H, C*H_a_*H_b_), 3.13–3.06 (dd, *J* = 15.18, 5.12 Hz, 1H, CH_a_*H_b_*), 3.64 (s, 3H, OC*H_3_*), 3.83–3.78 (t, *J* = 6.21 Hz, 1H, C*H*COOCH_3_), 5.35 (s, 1H, C*H*Ph), 7.08–6.96 (m, 2H, Ar-*H*), 7.29–7.23 (t, *J* = 8.27 Hz, 3H, Ar-*H*), 7.53–7.45 (dd, *J* = 15.89, 7.95 Hz, 3H, Ar-*H*), 10.59 (s, 1H, N*H*). ^13^C-NMR (DMSO-*d*_6_): δ 173.66, 142.39, 136.18, 133.76, 130.97, 130.45, 126.48, 120.90, 120.34, 118.40, 117.60, 111.05, 106.70, 53.44 (C1), 51.85 (C3), 51.57, 24.68. ESI-MS: m/z 343 (M^+^ + 2), m/z 341 (M^+^).

*(1R,3R)-1-(4-Bromophenyl)-2,3,4,9-tetrahydro-1H-β-carboline -3-carboxylic acid methyl ester* (**5e**). Orange solid. Yield: 47%. mp 222–224 °C. TLC, R_f_: 0.74. IR: 3375.1 (NH), 1737.3 (CO). ^1^H-NMR (DMSO-*d*_6_) δ: 2.91–2.82 (m, 1H, C*H_a_*H_b_), 3.09–3.02 (m, 1H, CH_a_*H_b_*), 3.72 (s, 3H, OC*H_3_*), 3.91–3.86 (dd, *J* = 10.93, 4.16 Hz, 1H, C*H*COOCH_3_), 5.23 (s, 1H, C*H*Ph), 7.05–6.94 (m, 2H, Ar-*H*), 7.24–7.21 (m, 1H, Ar-*H*), 7.34–7.31 (m, 2H, Ar-*H*), 7.46–7.43 (d, *J* = 7.49 Hz, 1H, Ar-*H*), 7.56–7.53 (m, 2H, Ar-*H*), 10.3 (s, 1H, N*H*). ^13^C-NMR (DMSO-*d*_6_): δ 172.52, 141.31, 136.29, 134.66, 130.96, 130.66, 126.34, 120.70, 120.60, 118.27, 117.37, 110.99, 106.91, 56.98 (C1), 56.06 (C3), 51.50, 25.16. ESI-MS: m/z 387 (M^+^ + 2), m/z 385 (M^+^).

*(1S,3R)-1-(4-Bromophenyl)-2,3,4,9-tetrahydro-1H-β-carboline-3-carboxylic acid methyl ester* (**5f**). Orange solid. Yield: 20%. mp 192–194 °C. TLC, R_f_: 0.43. IR: 3334.5 (NH), 1709.6 (CO). ^1^H-NMR (DMSO-*d*_6_) δ: 2.95–2.87 (dd, *J* = 15.21, 7.32 Hz, 1H, C*H_a_*H_b_), 3.10–3.04 (dd, *J* = 15.17, 5.14 Hz, 1H, CH_a_*H_b_*), 3.63 (s, 3H, OC*H_3_*), 3.80–3.75 (m, 1H, C*H*COOCH_3_), 5.32 (s, 1H, C*H*Ph), 7.06–6.97 (m, 2H, Ar-*H*), 7.24 (t, *J* = 7.37 Hz, 3H, Ar-*H*), 7.53–7.44 (dd, *J* = 18.82, 7.95 Hz, 3H, Ar-*H*), 10.61 (s, 1H, N*H*). ^13^C-NMR (DMSO-*d*_6_): δ 173.47, 142.26, 136.05, 133.62, 130.80, 130.26, 126.35, 120.72, 120.14, 118.22, 118.43, 110.88, 106.56, 53.29 (C1), 51.71 (C3), 51.37, 24.52. ESI-MS: m/z 387 (M^+^ + 2), m/z 385 (M^+^).

*(1S,3S)-1-(4-Bromophenyl)-2,3,4,9-tetrahydro-1H-β-carboline-3-carboxylic acid methyl ester* (**5g**). Orange Solid. Yield: 44.4%. mp 221–223 °C. TLC, R_f_: 0.74.IR: 3382.7 (NH), 1726.2 (CO). ^1^H-NMR (DMSO-*d*_6_) δ: 2.90–2.82 (m, 1H, C*H_a_*H_b_), 3.08–3.04 (m, 1H, CH_a_*H_b_*), 3.70 (s, 3H, OC*H_3_*), 3.89–3.85 (m, 1H, C*H*COOCH_3_), 5.20 (s, 1H, C*H*Ph), 7.03–6.95 (m, 2H, Ar-*H*), 7.32–7.21 (dd, *J* = 15.13, 7.26 Hz, 3H, Ar-*H*), 7.54–7.42 (m, 3H, Ar-*H*), 10.32 (s, 1H, N*H*). ^13^C-NMR (DMSO-*d*_6_): δ 172.63, 141.12, 136.45, 134.64, 131.21, 130.98, 126.45, 120.95, 120.91, 118.54, 117.65, 111.24, 107.03, 57.17 (C1), 56.15 (C3), 51.86, 25.21.ESI-MS: m/z 387 (M^+^ + 2), m/z 385 (M^+^).

*(1R,3S)-1-(4-Bromophenyl)-2,3,4,9-tetrahydro-1H-β-carboline-3-carboxylic acid methyl ester* (**5h**). Yellow solid. Yield: 33%. mp 192–194 °C. TLC, R_f_: 0.43. IR: 3333.3 (NH), 1709.4 (CO). ^1^H-NMR (DMSO-*d*_6_) δ: 2.97–2.90 (dd, *J* = 15.18, 7.38 Hz, 1H, C*H_a_*H_b_), 3.13–3.06 (dd, *J* = 15.18, 5.12 Hz, 1H, CH_a_*H_b_*), 3.64 (s, 3H, OC*H_3_*), 3.83–3.78 (t, *J* = 6.21 Hz, 1H, C*H*COOCH_3_), 5.35 (s, 1H, C*H*Ph), 7.08–6.96 (m, 2H, Ar-*H*), 7.29–7.23 (t, *J* = 8.27 Hz, 3H, Ar-*H*), 7.53–7.45 (dd, *J* = 15.89, 7.95 Hz, 3H, Ar-*H*), 10.59 (s, 1H, N*H*). ^13^C-NMR (DMSO-*d*_6_): δ 173.66, 142.39, 136.18, 133.76, 130.97, 130.45, 126.48, 120.90, 120.34, 118.40, 117.60, 111.05, 106.70, 53.44 (C1), 51.85 (C3), 51.57, 24.68. ESI-MS: m/z 387 (M^+^ + 2), m/z 385 (M^+^).

#### 2.1.3. General Procedure for the Preparation of Chloroethanone Intermediates

To a well-stirred solution of the appropriate β-carboline (**5a**–**5h**) and NaHCO_3_ (0.57 g, 6.89 mmol) in CHCl_3_ (40 mL) was added dropwise (1.1 mL, 13.69 mmol) chloroacetylchloride at 0 °C. The mixture was then stirred under N_2_ for 1 h. The mixture was then diluted with CH_2_Cl_2_, washed with a solution of NaHCO_3_, and dried over anhydrous Na_2_SO_4_. The filtrate was evaporated to dryness and the residue was then crystallized from diethyl ether.

#### 2.1.4. General Procedure for the Preparation of Compounds **6a**–**6p**, **7a**–**7p**

A solution of the appropriate chloroethanone derivative (1.2 mmol) in methanol (25 mL) was heated to reflux with the appropriate amine, namely ethanol amine, ethylenediamine, butanolamine, & 1,4-diaminobutane (2.4 mmol) under nitrogen atmosphere for 16 h, the reaction mixture was cooled to room temperature and then evaporated to dryness under reduced pressure. The residue was dissolved in ethyl acetate, and the organic layer was washed with water, dried over anhydrous MgSO_4_, filtered, and concentrated till dryness. The crude product was purified using column chromatography eluting with the CH_2_Cl_2_ & CH_3_OH solvent system or with the CH_2_Cl_2_, CH_3_OH, and triethylamine solvent system in the appropriate ratio.

*(6R,12aR)6-(4-Chloro-phenyl)-2-(2-hydroxy-ethyl)-2,3,6,7,12,12a-hexahydro-pyrazino[1’,2’:1,6]pyrido[3,4-b]indole-1,4-dione* (**6a**). Orange solid. Yield: 10%. mp 230–233 °C. TLC, R_f_: 0.39 (CH_2_Cl_2_/CH_3_OH, 97:3). IR: 3056.91 (OH), 2920.61 (NH), 1658.54, 1650.29 (CO). ^1^H-NMR (Acetone-*d*_6_) δ: 3.02 (ddd, *J* = 15.5, 11.9, 1.2 Hz, 1H, CH_a_C*H_b_*), 3.39 (ddd, *J* = 14.2, 6.3, 3.7 Hz, 1H, NCH_a_*H_b_*CH_2_), 3.54 (dd, *J* = 15.5, 4.3 Hz, 1H, C*H_a_*CH_b_ ), 3.71 (ddd, *J* = 14.1, 6.3, 3.9 Hz, 1H, NC*H_a_*H_b_CH_2_), 3.87–3.82 (m, 2H, C*H_2_*OH), 4.12 (d, *J* = 17.7 Hz, 1H, CH_a_*H_b_*C(O)N), 4.29 (dd, *J* = 21.3, 10.6 Hz, 2H, C*H_a_*H_b_C(O)N, CHC(O)), 6.99 (s, 1H, C*H*Ph), 7.19–7.14 (m, 3H, Ar-*H*), 7.25–7.21 (m, 2H, Ar-*H*), 7.27 (d, *J* = 12.9 Hz, 1H, Ar-*H*), 7.33 (d, *J* = 8.1 Hz, 1H, Ar-*H*), 7.52 (d, *J* = 7.8 Hz, 1H,Ar-*H*), 10.22 (s, 1H, N*H*). ESI-MS: m/z 411 (M^+^ + 2), m/z 409 (M^+^).

*(6R,12aR)-2-(2-Amino-ethyl)-6-(4-chloro-phenyl)-2,3,6,7,12,12a-hexahydro-pyrazino[1’,2’:1,6]pyrido[3,4-b]indole-1,4-dione* (**6b**). Yellow solid. Yield: 13%. mp 231–234 °C. TLC, R_f_: 0.54 (CH_2_Cl_2_/CH_3_OH/TEA, 95:4:1). IR: 3733.97, 3662.21 (NH_2_), 2968 (NH), 1663.54, 1654.29 (CO). ^1^H-NMR (Acetone-*d*_6_) δ: 2.97 (ddd, *J* = 15.4, 11.9, 1.5 Hz, 1H, CH_a_C*H_b_*), 3.37 (dd, *J* = 15.3, 4.1 Hz, 1H, C*H_a_*CH_b_), 3.45–3.41 (m, 1H, NCH_a_*H_b_*CH_2_), 3.59–3.51 (m, 2H, C*H_2_*NH_2_), 3.66–3.61 (m, 1H, NC*H_a_*H_b_CH_2_), 4.16 (dd, *J* = 11.8, 4.3 Hz, 1H, C*H*C(O)), 4.23 (dd, *J* = 17.8, 0.5 Hz, 1H, CH_a_*H_b_*C(O)N), 4.42 (dd, *J* = 17.8, 1.0 Hz, 1H, C*H_a_*H_b_C(O)N), 7.04 (s, 1H, C*H*Ph), 7.08 (ddd, *J* = 8.1, 7.1, 1.0 Hz, 1H, Ar-*H*), 7.15 (ddd, *J* = 8.2, 7.1, 1.2 Hz, 1H, Ar-*H*), 7.32–7.30 (m, 1H, Ar-*H*), 7.30–7.28 (m, 1H, Ar-*H*), 7.38 (d, *J* = 1.8 Hz, 2H, Ar*-H*), 7.4 (d, *J* = 1.3 Hz, 1H, Ar-*H*), 7.57 (d, *J* = 7.7 Hz, 1H, Ar-*H*), 10.22 (s, 1H, N*H*). ESI-MS: m/z 410 (M^+^ + 2), m/z 408 (M^+^). 

*(6R,12aR)-6-(4-Chloro-phenyl)-2-(4-hydroxy-butyl)-2,3,6,7,12,12a-hexahydropyrazino[1’,2’:1,6]pyrido[3,4-b]indole-1,4-dione* (**6c**). Orange solid, yield: 48%. mp 228–230 °C. TLC, R_f_: 0.31 (CH_2_Cl_2_/CH_3_OH, 97:3). IR: 3100.62 (OH), 2920 (NH), 1660.54, 1650 (CO). ^1^H-NMR (Acetone-*d*_6_) δ: 1.55–1.50 (m, 2H, CH_2_ C*H_2_*), 1.71–1.65 (m, 2H, C*H_2_*CH_2_), 3.13 (ddd, *J* = 16.0, 11.6, 1.3 Hz, 1H, CH_a_C*H_b_*), 3.46–3.40 (m, 1H, NCH_a_*H_b_*CH_2_), 3.55–3.51 (m, 1H, NC*H_a_*H_b_CH_2_), 3.58 (dd, *J* = 11.1, 6.3 Hz, 2H, C*H_2_*OH), 3.65 (dd, *J* = 16.2, 4.9 Hz, 1H, C*H_a_*CH_b_), 3.93 (d, *J* = 17.1 Hz, 1H, CH_a_*H_b_*C(O)N), 4.24 (dd, *J* = 17.1, 1.3 Hz, 1H, C*H_a_*H_b_C(O)N), 4.46 (dd, *J* = 11.5, 5.7 Hz, 1H, C*H*C(O)), 6.32 (s, 1H, C*H*Ph), 7.07–7.03 (m, 1H, Ar-*H*), 7.11–7.07 (m, 1H, Ar-*H*), 7.26–7.24 (m, 1H, Ar-*H*), 7.28–7.26 (m, 1H, Ar-*H*), 7.32–7.29 (m, 1H, Ar-*H*), 7.38–7.37 (m, 1H, Ar-*H*),7.40–7.39 (m, 1H, Ar-*H*), 7.61–7.58 (m, 1H, Ar-*H*), 10.20 (s, 1H, N*H*). ESI-MS: m/z 440 (M^+^ + 2), m/z 438 (M^+^). 

*(6R,12R)-2-(4-Amino-butyl)-6-(4-chloro-phenyl)-2,3,6,7,12,12a-hexahydro-pyrazino[1’,2’:1,6]pyrido[3,4-b]indole-1,4-dione* (**6d**). Yellow solid. Yield: 5%. mp 228–232 °C. TLC, R_f_: 0.35 (CH_2_Cl_2_/CH_3_OH/TEA, 93:5:2). IR: 3666.41, 3542.31 (NH_2_), 2930.4 (NH), 1661.4, 1652 (CO). ^1^H-NMR (DMSO-*d*_6_): δ 1.37–1.27 (m, 2H, CH_2_C*H_2_*), 1.57–1.47 (m, 2H, C*H_2_*CH_2_), 2.58–2.52 (m, 2H, C*H_2_*NH_2_), 2.99 (dd, *J* = 14.8, 11.2 Hz, 1H, CH_a_*H_b_*), 3.28 (dd, *J* = 15.1, 4.5 Hz, 1H, C*H_a_*H_b_), 3.52–3.40 (m, 2H, NC*H_a_H_b_*CH_2_), 4.08–3.88 (m, 2H, CHC(O), CH_a_*H_b_*C(O)N), 4.29 (d, *J* = 17.7 Hz, 1H, C*H_a_*H_b_C(O)N), 6.90 (s, 1H, C*H*Ph), 7.06–7.00 (m, 1H, Ar-*H*), 7.15–7.08 (m, 1H, Ar-*H*), 7.35–7.22 (m, 4H, Ar-*H*), 7.44 (d, *J* = 8.4 Hz, 1H, Ar-*H*), 7.57–7.51 (m, 1H, Ar-*H*), 11.07 (s, 1H, N*H*). ESI-MS: m/z 439 (M^+^ + 2), m/z 437 (M^+^).

*(6S,12aR)-6-(4-Chloro-phenyl)-2-(2-hydroxy-ethyl)-2,3,6,7,12,12a-hexahydro-pyrazino[1’,2’:1,6]pyrido[3,4-b]indole-1,4-dione* (**6e**). Orange solid. Yield: 26%. mp 202–206 °C. TLC, R_f_: 0.39, (CH_2_Cl_2_/CH_3_OH, 97:3). IR: 3056.91 (OH), 2920.61 (NH), 1658.54, 1650.29 (CO). ^1^H-NMR (DMSO-*d*_6_): δ 3.01 (dd, *J* = 15.8, 11.1 Hz, 1H, CH_a_*H_b_*), 3.42–3.33 (m, 3H, NC*H_a_H_b_*CH_2_, C*H_a_*H_b_), 3.56 (dd, *J* = 7.6, 3.7 Hz, 2H, C*H_2_*OH), 4.06 (dd, *J* = 11.7, 4.3 Hz, 1H, C*H*C(O)), 4.14 (d, *J* = 17.7 Hz, 1H, CH_a_*H_b_*C(O)N), 4.34 (d, *J* = 17.7 Hz, 1H, C*H_a_*H_b_C(O)N), 6.91 (s, 1H, C*H*Ph), 7.03 (t, *J* = 7.4 Hz, 1H, Ar-*H*), 7.12 (t, *J* = 7.3 Hz, 1H, Ar-*H*), 7.24 (d, *J* = 8.5 Hz, 2H, Ar-*H*), 7.26–7.24 (m, 1H, Ar-*H*), 7.33 (d, *J* = 8.0 Hz, 1H, Ar-*H*), 7.47–7.42 (m, 2H, Ar-*H*), 7.53 (d, *J* = 7.4 Hz, 1H, Ar-*H*), 11.05 (s, 1H, N*H*). ESI-MS: m/z 411 (M^+^ + 2), m/z 409 (M^+^).

*(6S,12aR)-2-(2-Amino-ethyl)-6-(4-chloro-phenyl)-2,3,6,7,12,12a-hexahydro-pyrazino[1’,2’:1,6]pyrido[3,4-b]indole-1,4-dione* (**6f**). Orange solid. Yield: 10%. mp 200–204 °C. TLC, R_f_: 0.54, (CH_2_Cl_2_/CH_3_OH/TEA, 95:4:1). IR: 3733.97, 3662.21 (NH_2_), 2968 (NH), 1660.64, 1652.21 (CO). ^1^H-NMR (DMSO-*d*_6_): δ 2.75 (t, *J* = 7.9 Hz, 2H, C*H_2_*NH_2_), 3.04 (dd, *J* = 15.2, 11.3 Hz, 1H,CH_a_*H_b_*), 3.49–3.39 (m, 2H, NC*H_a_H_b_*CH_2_), 4.10 (d, *J* = 17.3 Hz, 2H, CH_a_*H_b_*C(O)N, C*H*C(O)), 4.34 (d, *J* = 18.0 Hz, 1H, C*H_a_*H_b_C(O)N), 6.91 (s, 1H, C*H*Ph), 7.04 (t, *J* = 7.3 Hz, 1H, Ar-*H*), 7.12 (t, *J* = 7.6 Hz, 1H, Ar-*H*), 7.25 (d, *J* = 8.1 Hz, 2H, Ar-*H*), 7.34 (d, *J* = 7.9 Hz, 1H, Ar-*H*), 7.44 (d, *J* = 8.4 Hz, 2H, Ar-*H*), 7.53 (d, *J* = 7.5 Hz, 1H, Ar-*H*), 11.06 (s, 1H, N*H*). ESI-MS: m/z 410 (M^+^ + 2), m/z 408 (M^+^).

*(6S,12aR)-6-(4-Chloro-phenyl)-2-(4-hydroxy-butyl)-2,3,6,7,12,12a-hexahydropyrazino[1’,2’:1,6]pyrido[3,4-b]indole-1,4-dione* (**6g**). Orange solid. Yield: 64%. mp 200–203 °C. TLC, R_f_: 0.31, (CH_2_Cl_2_/CH_3_OH, 97:3). IR: 3100.62 (OH), 2920 (NH), 1660.54, 1650 (CO). ^1^H-NMR (DMSO-*d*_6_): δ 1.43–1.33 (m, 2H, CH_2_C*H_2_*), 1.59–1.48 (m, 2H, C*H_2_*CH_2_), 2.99 (dd, *J* = 15.5, 11.6 Hz, 1H,CH_a_*H_b_*), 3.19–3.12 (m, 1H, NCH_a_*H_b_*CH_2_), 3.40 (dd, *J* = 11.7, 6.1 Hz, 2H, C*H_2_*OH), 3.54–3.44 (m, 1H, NC*H_a_*H_b_CH_2_), 4.07–3.96 (m, 2H, CH_a_*H_b_*C(O)N, C*H*C(O)), 4.30 (d, *J* = 17.6 Hz, 1H, C*H_a_*H_b_C(O)N), 6.90 (s, 1H, C*H*Ph), 7.03 (t, *J* = 7.4 Hz, 1H, Ar-*H*), 7.12 (t, *J* = 7.5 Hz, 1H, Ar-*H*), 7.23 (d, *J* = 8.4 Hz, 2H, Ar-*H*), 7.33 (d, *J* = 7.9 Hz, 1H, Ar-*H*), 7.44 (d, *J* = 8.4 Hz, 2H, Ar-*H*), 7.53 (d, *J* = 7.7 Hz, 1H, Ar-*H*), 11.08 (s, 1H, N*H*). ESI-MS: m/z 440 (M^+^ + 2), m/z 438 (M^+^).

*(6S,12R)-2-(4-Amino-butyl)-6-(4-chloro-phenyl)-2,3,6,7,12,12a-hexahydro-pyrazino[1’,2’:1,6]pyrido[3,4-b]indole-1,4-dione* (**6h**). Yellow solid. Yield: 29%. mp 200–202 °C. TLC, R_f_: 0.35, (CH_2_Cl_2_/CH_3_OH/TEA, 93:5:2). IR: 3666.41, 3542.31 (NH_2_), 2930.4 (NH), 1662.3, 1654 (CO). ^1^H-NMR (Acetone-*d*_6_): δ 1.61–1.55 (m, 2H, CH_2_C*H_2_*), 1.69–1.63 (m, 2H,C*H_2_*CH_2_), 2.99 (ddd, *J* = 15.4, 11.9, 1.5 Hz, 1H, CH_a_*H_b_*), 3.20 (t, *J* = 6.7 Hz, 2H, C*H_2_*NH_2_), 3.37 (dd, *J* = 15.4, 4.2 Hz, 1H, C*H_a_*H_b_), 3.60–3.57 (m, 1H, NCH_a_*H_b_*CH_2_), 3.63–3.61 (m, 1H, NC*H_a_*H_b_CH_2_), 4.03 (d, *J* = 17.6 Hz, 1H, CH_a_*H_b_*C(O)N), 4.17 (dd, *J* = 11.8, 4.1 Hz, 1H, CHC(O)), 4.30 (dd, *J* = 17.6, 1.1 Hz, 1H, C*H_a_*H_b_C(O)N), 7.04 (s, 1H, C*H*Ph), 7.08 (ddd, *J* = 8.0, 7.1, 1.0 Hz, 1H, Ar-*H*), 7.15 (ddd, *J* = 8.2, 7.1, 1.2 Hz, 1H, Ar-*H*), 7.30–7.28 (m, 2H, Ar-*H*), 7.38–7.36 (m, 2H, Ar-*H*), 7.40–7.38 (m, 1H, Ar-*H*), 7.58–7.55 (m, 1H, Ar-*H*), 10.26 (s, 1H, NH). ESI-MS: m/z 439 (M^+^ + 2), m/z 437 (M^+^).

*(6S,12aS)-6-(4-Chloro-phenyl)-2-(2-hydroxy-ethyl)-2,3,6,7,12,12a-hexahydro-pyrazino[1’,2’:1,6]pyrido[3,4-b]indole-1,4-dione* (**6i**). Yellow solid. Yield: 40%. mp 231–233 °C, TLC, R_f_: 0.39, (CH_2_Cl_2_/CH_3_OH, 97:3). IR: 3056.91 (OH), 2920.61 (NH), 1658.54, 1650.29 (CO). ^1^H-NMR (Acetone-*d*_6_): δ 3.01 (ddd, *J* = 15.4, 11.9, 1.5 Hz, 1H, CH_a_*H_b_*), 3.38 (dd, *J* = 15.4, 4.3 Hz, 1H, C*H_a_*H_b_), 3.47 (ddd, *J* = 13.8, 6.0, 4.9 Hz, 1H, NCH_a_*H_b_*CH_2_), 3.63–3.57 (m, 1H, NC*H_a_*H_b_CH_2_), 3.75 (ddd, *J* = 10.4, 5.4, 2.7 Hz, 2H, C*H_2_*OH), 4.24–4.17 (m, 2H, C*H*C(O), CH_a_*H_b_*C(O)N), 4.40 (dd, *J* = 17.7, 1.1 Hz, 1H, C*H_a_*H_b_C(O)N), 7.04 (s, 1H, C*H*Ph), 7.08 (ddd, *J* = 8.0, 7.1, 1.0 Hz, 1H, Ar-*H*), 7.15 (ddd, *J* = 8.2, 7.1, 1.2 Hz, 1H, Ar-*H*), 7.30–7.28 (m, 1H, Ar-*H*), 7.32–7.31 (m, 1H, Ar-*H*), 7.38–7.36 (m, 2H, Ar-*H*), 7.39 (d, *J* = 1.6 Hz, 1H, Ar-*H*), 7.57 (d, *J* = 7.9 Hz, 1H, Ar-*H*), 10.15 (s, 1H, N*H*). ). ^13^C-NMR (Acetone-*d*_6_): δ 166, 163.3 (NCO), 139.28, 137.8, 134.54, 131.21, 130.85, 129.52, 127.39, 122.96, 120.22, 112.21, 109.56, 60.39 (NC(O)*C*H_a_H_b_), 53.55 (C6), 51.83 (C12), 51.6 (N*C*H_a_H_b_CH_2_), 49.48 (CH_2_OH), 27.84 (C*C*H_a_H_b_). ESI-MS: m/z 411 (M^+^ + 2), m/z 409 (M^+^).

*(6S,12aS)-2-(2-Amino-ethyl)-6-(4-chloro-phenyl)-2,3,6,7,12,12a-hexahydro-pyrazino[1’,2’:1,6]pyrido[3,4-b]indole-1,4-dione* (**6j**). Yellow solid. Yield: 30%. mp 231–233 °C, TLC, R_f_: 0.54, (CH_2_Cl_2_/CH_3_OH/TEA, 95:4:1). IR: 3733.97, 3662.21 (NH_2_), 2968 (NH), 1657.4, 1650 (CO). ^1^H-NMR (Acetone-*d*_6_): δ 2.97 (ddd, *J* = 15.4, 11.9, 1.5 Hz, 1H, CH_a_*H_b_*), 3.38 (dd, *J* = 15.4, 4.1 Hz, 1H, C*H_a_*H_b_), 3.47–3.40 (m, 2H, C*H_2_*NH_2_), 3.56–3.51 (m, 1H, NCH_a_*H_b_*CH_2_), 3.66–3.61 (m, 1H, NC*H_a_*H_b_CH_2_), 4.16 (dd, *J* = 11.9, 4.2 Hz, 1H, CHC(O)), 4.23 (d, *J* = 17.4 Hz, 1H, CH_a_*H_b_*C(O)N), 4.41 (dd, *J* = 17.8, 1.1 Hz, 1H, C*H_a_*H_b_C(O)N), 7.04 (s, 1H, C*H*Ph), 7.08 (ddd, *J* = 8.0, 7.1, 1.0 Hz, 1H, Ar-*H*), 7.15 (ddd, *J* = 8.2, 7.1, 1.2 Hz, 1H, Ar-*H*), 7.29–7.28 (m, 1H, Ar-*H*), 7.31–7.30 (m, 1H, Ar-*H*), 7.39–7.36 (m, 2H, Ar-*H*), 7.40–7.39 (m, 1H, Ar-*H*), 7.58–7.55 (m, 1H, Ar-*H*), 10.23 (s, 1H, N*H*).^13^C-NMR (Acetone-*d*_6_): δ 165.62, 163.44 (NCO), 139.3, 137.8, 134.52, 131.16, 130.88, 129.52, 127.39, 122.94, 120.19, 119.05, 112.17, 109.55, 53.57 (C6), 51.79 (C12), 49.76 (NC(O)*C*H_a_H_b_), 47.99 (N*C*H_a_H_b_CH_2_), 47.01 (CH_2_NH_2_), 27.78 (CCH_a_H_b_). ESI-MS: m/z 410 (M^+^ + 2), m/z 408 (M^+^).

*(6S,12aS)-6-(4-Chloro-phenyl)-2-(4-hydroxy-butyl)-2,3,6,7,12,12a-hexahydropyrazino[1’,2’:1,6]pyrido[3,4-b]indole-1,4-dione* (**6k**). Yellow solid. Yield: 31%. mp: 227–229 °C. TLC, R_f_: 0.31, (CH_2_Cl_2_/CH_3_OH, 97:3). IR: 3100.62 (OH), 2920 (NH), 1660.54, 1650 (CO). ^1^H-NMR (Acetone-*d*_6_): δ 1.56–1.51 (m, 2H, CH_2_C*H_2_*), 1.71–1.64 (m, 2H, C*H_2_*CH_2_), 3.13 (ddd, *J* = 16.0, 11.6, 1.3 Hz, 1H, CH_a_*H_b_*), 3.46–3.40 (m, 1H, NCH_a_*H_b_*CH_2_), 3.52–3.49 (m, 1H, NC*H_a_*H_b_CH_2_), 3.58 (dd, *J* = 11.7, 6.6 Hz, 2H, C*H_2_*OH), 3.65 (dd, *J* = 15.8, 4.5 Hz, 1H, C*H_a_*H_b_), 3.93 (d, *J* = 17.1 Hz, 1H, CH_a_*H_b_*C(O)N), 4.24 (dd, *J* = 17.1, 1.4 Hz, 1H, C*H_a_*H_b_C(O)N), 4.47 (ddd, *J* = 11.6, 4.7, 1.1 Hz, 1H, C*H*C(O)), 6.32 (s, 1H, C*H*Ph), 7.07–7.03 (m, 1H, Ar-*H*), 7.10–7.07 (m, 1H. Ar-*H*), 7.26–7.25 (m, 1H, Ar-*H*), 7.28–7.26 (m, 1H, Ar-*H*), 7.30 (ddd, *J* = 8.0, 1.3, 0.8 Hz, 1H, Ar-*H*), 7.38–7.37 (m, 1H, Ar-*H*), 7.40–7.39 (m, 1H, Ar-*H*), 7.60 (ddd, *J* = 7.6, 1.0, 0.4 Hz, 1H, Ar-*H*), 10.21 (s, 1H, N*H*). ^13^C-NMR (Acetone-*d*_6_): δ 168.17, 167.37 (NCO), 143, 137.87, 134.32, 133.06, 129.55, 129.15, 127.25, 122.58, 120.17, 119.10, 112.16, 106.87, 62.07 (C6), 56.78 (C12), 56.54 (NC(O)*C*H_a_H_b_), 50.74 (N*C*H_a_H_b_CH_2_), 46.34 (*C*H_2_OH), 30.7 (*C*H_2_CH_2_), 24.315 (C*C*H_a_H_b_), 24.2 (CH_2_*C*H_2_). ESI-MS: m/z 440 (M^+^ + 2), m/z 438 (M^+^).

*(6S,12S)-2-(4-Amino-butyl)-6-(4-chloro-phenyl)-2,3,6,7,12,12a-hexahydro-pyrazino[1’,2’:1,6]pyrido[3,4-b]indole-1,4-dione* (**6l**). Yellow solid. Yield: 27%. mp: 229–231 °C. TLC, R_f_: 0.35, (CH_2_Cl_2_/CH_3_OH/TEA, 93:5:2). IR: 3666.41, 3542.31 (NH_2_), 2930.4 (NH), 1660.7, 1653.5 (CO). ^1^H-NMR (Acetone-*d*_6_): δ 1.62–1.55 (m, 2H, CH_2_C*H_2_*), 1.67 (ddd, *J* = 11.5, 6.9, 4.0 Hz, 2H, C*H_2_*CH_2_), 3.11 (ddd, *J* = 15.9, 11.6, 1.1 Hz, 1H, CH_a_*H_b_*), 3.22 (t, *J* = 6.7 Hz, 2H, C*H_2_*NH_2_), 3.44 (dt, *J* = 13.7, 7.0 Hz, 1H, NCH_a_*H_b_*CH_2_), 3.62–3.58 (m, 1H, NC*H_a_*H_b_CH_2_), 3.66 (dd, *J* = 11.6, 5.8 Hz, 1H, C*H_a_*H_b_), 3.92 (d, *J* = 17.0 Hz, 1H, CH_a_*H_b_*C(O)N), 4.24 (dd, *J* = 17.0, 1.3 Hz, 1H, C*H_a_*H_b_C(O)N), 4.44 (dd, *J* = 11.5, 5.6 Hz, 1H, C*H*C(O)), 6.35 (s, 1H, C*H*Ph), 7.05–7.02 (m, 1H, Ar-*H*), 7.10–7.06 (m, 1H, Ar-*H*), 7.23 (t, *J* = 2.3 Hz, 1H, Ar-*H*), 7.26–7.24 (m, 1H, Ar-*H*), 7.34–7.31 (m, 1H, Ar-*H*), 7.43–7.39 (m, 2H, Ar-*H*), 7.60–7.57 (m, 1H, Ar-*H*), 10.51 (s, 1H, NH). ^13^C-NMR (Acetone-*d*_6_): δ 168.14, 167.4 (NCO), 143.06, 137.72, 134.28, 132.98, 129.52, 129.11, 127.2, 122.49, 120.08, 119.05, 112.17, 106.69, 56.77 (C6), 56.41 (NC(O)CH_a_H_b_), 51.23 (C12), 50.74 (N*C*H_a_H_b_CH_2_), 46.23 (CH_2_NH_2_), 28.63 (*C*H_2_CH_2_), 25.66 (C*C*H_a_H_b_), 24.17 (CH_2_*C*H_2_). ESI-MS: m/z 439 (M^+^ + 2), m/z 437 (M^+^).

*(6R,12aS)-6-(4-Chloro-phenyl)-2-(2-hydroxy-ethyl)-2,3,6,7,12,12a-hexahydro-pyrazino[1’,2’:1,6]pyrido[3,4-b]indole-1,4-dione* (**6m**). White solid. Yield: 20%. mp: 202–206 °C. TLC, R_f_: 0.39, (CH_2_Cl_2_/CH_3_OH, 97:3). IR: 3056.91 (OH), 2920.61 (NH), 1658.54, 1650.29 (CO). ^1^H-NMR (DMSO-*d*_6_): δ 2.99 (ddd, *J* = 15.6, 11.9, 1.5 Hz, 1H, CH_a_C*H_b_*), 3.49 (ddd, *J* = 14.3, 6.1, 3.8 Hz, 1H, NCH_a_*H_b_*CH_2_), 3.58 (dd, *J* = 15.5, 4.2 Hz, 1H, C*H_a_*H_b_), 3.73 (ddd, *J* = 14.2, 6.2, 4.0 Hz, 1H, NC*H_a_*H_b_CH_2_), 3.89 (dd, *J* = 9.5, 5.8 Hz, 2H, C*H_2_*OH), 4.16 (d, *J* = 17.5 Hz, 1H, CH_a_*H_b_*C(O)N), 4.35–4.30 (m, 2H,C*H*C(O), C*H_a_*H_b_C(O)N), 7.05 (s, 1H, C*H*Ph), 7.21–7.17 (m, 1H, Ar-*H*), 7.24 (t, *J* = 1.9 Hz, 1H, Ar-*H*), 7.26 (d, *J* = 2.2 Hz, 2H, Ar-*H*), 7.32–7.30 (m, 2H, Ar-*H*), 7.34 (d, *J* = 8.1 Hz, 1H, Ar-*H*), 7.55 (d, *J* = 7.4 Hz, 1H, Ar-*H*). ESI-MS: m/z 411(M^+^ + 2), m/z 409 (M^+^).

*(6R,12aS)-2-(2-Amino-ethyl)-6-(4-chloro-phenyl)-2,3,6,7,12,12a-hexahydro-pyrazino[1’,2’:1,6]pyrido[3,4-b]indole-1,4-dione* (**6n**). White solid. Yield: 48%. mp: 200–204 °C. TLC, R_f_: 0.54, (CH_2_Cl_2_/CH_3_OH/TEA, 95:4:1). IR: 3733.97, 3662.21 (NH_2_), 2968 (NH), 1656.7, 1648.11 (CO). ^1^H-NMR (Acetone-*d*_6_): δ 2.96 (ddd, *J* = 15.4, 11.9, 1.5 Hz, 1H, CH_a_C*H_b_*), 3.37 (dd, *J* = 15.4, 4.1 Hz, 1H, C*H_a_*CH_b_), 3.46–3.39 (m, 2H, C*H_2_*NH_2_), 3.54–3.49 (m, 1H, NCH_a_*H_b_*CH_2_), 3.65–3.60 (m, 1H, NC*H_a_*H_b_CH_2_), 4.16 (dd, *J* = 11.8, 4.2 Hz, 1H, CHC(O), 4.22 (d, *J* = 17.8 Hz, 1H, CH_a_*H_b_*C(O)N), 4.40 (dd, *J* = 17.8, 1.0 Hz, 1H, C*H_a_*H_b_C(O)N), 7.03 (s, 1H, C*H*Ph), 7.07 (ddd, *J* = 8.0, 7.1, 1.0 Hz, 1H, Ar-*H*), 7.14 (ddd, *J* = 8.2, 7.1, 1.2 Hz, 1H, Ar-*H*), 7.30–7.26 (m, 2H, Ar-*H*), 7.38–7.36 (m, 2H, Ar-*H*), 7.39 (dd, *J* = 2.9, 2.1 Hz, 1H, Ar-*H*), 7.57–7.54 (m, 1H, Ar-*H*), 10.27 (s, 1H, N*H*). ESI-MS: m/z 410 (M^+^ + 2), m/z 408 (M^+^).

*(6R,12aS)-6-(4-Chloro-phenyl)-2-(4-hydroxy-butyl)-2,3,6,7,12,12a-hexahydropyrazino[1’,2’:1,6]pyrido[3,4-b]indole-1,4-dione* (**6o**). Orange solid. Yield: 44%. mp: 199–203 °C. TLC, R_f_: 0.31, (CH_2_Cl_2_/CH_3_OH, 97:3). IR: 3100.62 (OH), 2920(NH), 1660.54, 1650 (CO). ^1^H-NMR (DMSO-*d*_6_): δ 1.44–1.35 (m, 2H, C*H_2_*CH_2_), 1.55 (dt, *J* = 7.4, 6.2 Hz, 2H, CH_2_C*H_2_*), 2.99 (m 1H, CH_a_*H_b_*), 3.43–3.37 (m, 2H, C*H_2_*OH), 3.48 (dd, *J* = 13.8, 7.7 Hz, 1H, NC*H_a_*H_b_CH_2_), 4.02 (d, *J* = 17.7 Hz, 2H, C*H*C(O), CH_a_*H_b_*C(O)N), 4.29 (d, *J* = 17.4 Hz, 1H, C*H_a_*H_b_C(O)N), 6.91 (s, 1H, C*H*Ph), 7.04 (t, *J* = 7.5 Hz, 1H, Ar-*H*), 7.13 (t, *J* = 7.4 Hz, 1H, Ar-*H*), 7.25 (d, *J* = 8.3 Hz, 2H, Ar-*H*), 7.34 (d, *J* = 8.1 Hz, 1H, Ar-*H*), 7.44 (d, *J* = 8.5 Hz, 2H, Ar-*H*), 7.53 (d, *J* = 7.5 Hz, 1H, Ar-*H*), 11.12 (s, 1H, NH). ESI-MS: m/z 440 (M^+^ + 2), m/z 438 (M^+^).

*(6R,12S)-2-(4-Amino-butyl)-6-(4-chloro-phenyl)-2,3,6,7,12,12a-hexahydro-pyrazino[1’,2’:1,6]pyrido[3,4-b]indole-1,4-dione* (**6p**). White solid. Yield: 50%. mp: 199–201 °C. TLC, R_f_: 0.35, (CH_2_Cl_2_/CH_3_OH/TEA, 93:5:2). IR: 3666.41, 3542.31 (NH_2_), 2930.4 (NH), 1663.5, 1652.6 (CO). ^1^H-NMR (Acetone-*d*_6_): δ 1.34 (t, *J* = 7.3 Hz, 2H, CH_2_C*H_2_*), 1.47 (t, *J* = 7.3 Hz, 2H, C*H_2_*CH_2_), 3.11–3.05 (m, 1H, CH_a_*H_b_*), 3.37 (dd, *J* = 15.4, 4.3 Hz, 1H, C*H_a_*H_b_), 3.53–3.51 (m, 1H, NCH_a_*H_b_*CH_2_), 3.59 (t, *J* = 2.8 Hz, 2H, C*H_2_*NH_2_), 3.62–3.60 (m, 1H, NC*H_a_*H_b_CH_2_), 4.06 (d, *J* = 17.6 Hz, 1H, CH_a_*H_b_*C(O)N), 4.17 (dd, *J* = 11.8, 4.2 Hz, 1H, C*H*C(O), 4.38 (d, *J* = 17.4 Hz, 1H,C*H_a_*H_b_C(O)N), 7.05 (s, 1H, C*H*Ph), 7.08 (dd, *J* = 11.0, 4.0 Hz, 1H, Ar-*H*), 7.14 (dd, *J* = 11.1, 4.1 Hz, 1H, Ar-*H*), 7.30 (t, *J* = 2.1 Hz, 1H, Ar-*H*), 7.32 (d, *J* = 2.2 Hz, 1H, Ar-*H*), 7.39–7.36 (m, 2H, Ar-*H*), 7.41–7.39 (m, 1H, Ar-*H*), 7.57 (d, *J* = 7.9 Hz, 1H, Ar-*H*), 10.31 (s, 1H, NH). ESI-MS: m/z 439 (M^+^ + 2), m/z 437 (M^+^).

*(6R,12R)-6-(4-Bromo-phenyl)-2-(2-hydroxy-ethyl)-2,3,6,7,12,12a-hexahydro-pyrazino[1’,2’:1,6]pyrido[3,4-b]indole-1,4-dione* (**7a**). Orange solid. Yield: 36.7%. mp: 233–235 °C. TLC, R_f_: 0.38, (CH_2_Cl_2_/CH_3_OH, 97:3). IR: 3056.91 (OH), 2920.61 (NH), 1656.8, 1650.29 (CO). ^1^H-NMR (DMSO-*d*_6_): δ 3.00 (dd, *J* = 15.3, 11.1 Hz, 1H, CH_a_*H_b_*), 3.41–3.36 (m, 1H, NCH_a_*H_b_*CH_2_), 3.60–3.51 (m, 2H, C*H_2_*OH), 3.50 (dd, *J* = 13.9, 7.0 Hz, 1H, NC*H_a_*H_b_NC(O)), 4.18–4.00 (m, 2H, CH_a_*H_b_*C(O)N, C*H*C(O)), 4.33 (d, *J* = 18.1 Hz, 1H, C*H_a_*H_b_C(O)N), 6.89 (s, 1H, C*H*Ph), 7.07–6.99 (m, 1H, Ar-*H*), 7.14–7.08 (m, 1H, Ar-*H*), 7.18 (d, *J* = 8.7 Hz, 2H, Ar-*H*), 7.33 (d, *J* = 7.8 Hz, 1H, Ar-*H*), 7.53 (d, *J* = 7.9 Hz, 2H, Ar-*H*), 7.58 (d, *J* = 7.8 Hz, 1H, Ar-*H*), 11.03 (s, 1H, NH). ESI-MS: m/z 456 (M^+^ + 2), m/z 454 (M^+^).

*(6R,12R)-2-(2-Amino-ethyl)-6-(4-bromo-phenyl)-2,3,6,7,12,12a-hexahydro-pyrazino[1’,2’:1,6]pyrido[3,4-b]indole-1,4-dione* (**7b**). Yellow solid. Yield: 12.2%. mp: 232–236 °C. TLC, R_f_: 0.49, (CH_2_Cl_2_/CH_3_OH/TEA, 95:4:1). IR: 3733.97, 3662.21 (NH_2_), 2968 (NH), 1659.2, 1651.4 (CO). ^1^H-NMR (Acetone-*d*_6_): δ 2.96 (ddd, *J* = 15.4, 11.9, 1.5 Hz, 1H, CH_a_*H_b_*), 3.37 (dd, *J* = 15.4, 4.2 Hz, 1H, C*H_a_*H_b_), 3.47–3.40 (m, 2H, C*H_2_*NH_2_), 3.55–3.50 (m, 1H, NCH_a_*H_b_*CH_2_), 3.66–3.61 (m, 1H, NC*H_a_*H_b_CH_2_), 4.16 (dd, *J* = 11.8, 4.2 Hz, 1H, CHC(O)), 4.23 (d, *J* = 17.8 Hz, 1H, CH_a_*H_b_*C(O)N), 4.41 (dd, *J* = 17.8, 1.1 Hz, 1H, C*H_a_*H_b_C(O)N), 7.03 (s, 1H, C*H*Ph), 7.08 (ddd, *J* = 8.0, 7.1, 1.0 Hz, 1H, Ar-*H*), 7.16–7.13 (m, 1H, Ar-*H*), 7.25–7.22 (m, 2H, Ar-*H*), 7.40–7.38 (m, 1H, Ar-*H*), 7.55–7.52 (m, 2H, Ar-*H*), 7.56 (d, *J* = 7.8 Hz, 1H, Ar-*H*). ^13^C-NMR (Acetone-*d_6_*): 165.2, 163.45 (NCO), 139.76, 137.73, 132.52, 131.48, 130.69, 127.34, 122.92, 120.18, 119, 112, 109, 53.59 (C6), 51.79 (C12), 49.76 (NC(O)*C*H_a_H_b_), 47.99 (NCH_a_H_b_), 46.92 (*C*H_2_NH_2_), 27.79 (C*C*H_a_H_b_).ESI-MS: m/z 455 (M^+^ + 2), m/z 453 (M^+^).

*(6R,12R)-6-(4-Bromo-phenyl)-2-(4-hydroxy-butyl)-2,3,6,7,12,12a-hexahydropyrazino[1’,2’:1,6]pyrido[3,4-b]indole-1,4-dione* (**7c**). Orange solid. Yield: 55.6%. mp: 234–237 °C. TLC, R_f_: 0.32, (CH_2_Cl_2_/CH_3_OH, 97:3). IR: 3100.62 (OH), 2920 (NH), 1680, 1665.4 (CO). ^1^H-NMR (Acetone-*d*_6_): δ 1.56–1.49 (m, 2H, CH_2_C*H_2_*), 1.71–1.64 (m, 2H, C*H_2_*CH_2_), 3.13 (ddd, *J* = 16.0, 11.6, 1.3 Hz, 1H, CH_a_*H_b_*), 3.46–3.40 (m, 1H, NCH_a_*H_b_*CH_2_), 3.61–3.56 (m, 3H, NC*H_a_*H_b_CH_2_, C*H_2_*OH), 3.65 (dd, *J* = 16.1, 4.8 Hz, 1H, C*H_a_*H_b_), 3.93 (d, *J* = 17.1 Hz, 1H, CH_a_*H_b_*C(O)N), 4.24 (dd, *J* = 17.1, 1.4 Hz, 1H, C*H_a_*H_b_C(O)N), 4.46 (ddd, *J* = 11.5, 4.6, 1.0 Hz, 1H, CHC(O)), 6.30 (s, 1H, C*H*Ph), 7.07–7.03 (m, 1H, Ar-*H*), 7.10–7.07 (m, 1H, Ar-*H*), 7.31–7.29 (m, 1H, Ar-*H*), 7.35–7.32 (m, 2H, Ar-*H*), 7.43–7.40 (m, 2H, Ar-*H*), 7.60 (d, *J* = 7.6 Hz, 1H, Ar-*H*), 10.20 (s, 1H, NH). ^13^C-NMR (Acetone-d_6_): 168.16, 167.34 (NCO), 143.5, 137.88, 134.25, 132.14, 129.91, 127.25, 122.59, 121.15, 120.17, 119.11, 112.15, 106.89, 62.07 (C6), 56.774 (C12), 56.6 (NC(O)*C*H_a_H_b_), 50.73 (N*C*H_a_H_b_), 46.34 (CH_2_OH), 30.70 (*C*H_2_CH_2_), 24.3 (CCH_a_H_b_), 24.2 (CH_2_*C*H_2_).ESI-MS: m/z 484 (M^+^ + 2), m/z 482 (M^+^).

*(6R,12R)-2-(4-Amino-butyl)-6-(4-bromo-phenyl)-2,3,6,7,12,12a-hexahydro-pyrazino[1’,2’:1,6]pyrido[3,4-b]indole-1,4-dione* (**7d**). Yellow solid. Yield: 38%. mp: 235–238 °C. TLC, R_f_: 0.37, (CH_2_Cl_2_/CH_3_OH/TEA, 93:5:2). IR: 3666.41, 3542.31 (NH_2_), 2930.4 (NH), 1669.2, 1660.1 (CO). ^1^H-NMR (Acetone-*d*_6_): δ 1.57 (ddt, *J* = 9.3, 7.1, 4.5 Hz, 2H, C*H_2_*CH_2_), 1.70–1.63 (m, 2H, CH_2_C*H_2_*), 2.98 (ddd, *J* = 15.4, 11.9, 1.5 Hz, 1H, CH_a_*H_b_*), 3.19 (t, *J* = 6.7 Hz, 2H, C*H_2_*NH_2_), 3.30 (dt, *J* = 11.6, 7.0 Hz, 1H, NCH_a_*H_b_*CH_2_), 3.37 (dd, *J* = 15.4, 4.2 Hz, 1H, C*H_a_*H_b_), 3.60–3.55 (m, 1H, NC*H_a_*H_b_CH_2_), 4.04 (d, *J* = 17.6 Hz, 1H, CH_a_*H_b_*C(O)N), 4.17 (dd, *J* = 11.9, 4.2 Hz, 1H, CHC(O)), 4.30 (dd, *J* = 17.6, 1.0 Hz, 1H, C*H_a_*H_b_C(O)N), 7.03(s, 1H, C*H*Ph), 7.09–7.06 (m, 1H, Ar-*H*), 7.16–7.13 (m, 1H, Ar-*H*), 7.25–7.22 (m, 2H, Ar-*H*), 7.40–7.38 (m, 1H, Ar-*H*), 7.54–7.51 (m, 2H, Ar-*H*), 7.56 (d, *J* = 7.8 Hz, 1H, Ar-*H*), 10.34 (s, 1H, NH). ^13^C-NMR (Acetone-*d*_6_): 165.53, 163.4 (NCO), 139.74, 137.66, 132.51, 131.48, 130.69, 127.34, 122.91, 122.7, 120.17, 119, 112, 109, 53.64 (C6), 51.89 (C12), 51.32 (NC(O)*C*H_a_H_b_), 49.89 (NCH_a_H_b_), 46.14 (CH_2_NH_2_), 28.73 (*C*H_2_CH_2_), 27.85 (C*C*H_a_H_b_), 25.12 (CH_2_*C*H_2_). ESI-MS: m/z 483 (M^+^ + 2), m/z 481(M^+^).

*(6S,12R)-6-(4-Bromo-phenyl)-2-(2-hydroxy-ethyl)-2,3,6,7,12,12a-hexahydro-pyrazino[1’,2’:1,6]pyrido[3,4-b]indole-1,4-dione* (**7e**). Yellow solid. Yield: 55%. mp: 206–208 °C. TLC, R_f_: 0.38, (CH_2_Cl_2_/CH_3_OH, 97:3). IR: 3056.91 (OH), 2920.61(NH), 1656.8, 1650.29 (CO). ^1^H-NMR (DMSO-*d*_6_): δ 3.00 (dd, *J* = 15.3, 11.8 Hz, 1H, CH_a_*H_b_*), 3.29 (dd, *J* = 15.7, 4.3 Hz, 1H, C*H_a_*H_b_), 3.57 (dd, *J* = 10.6, 4.9 Hz, 2H, C*H_2_*OH), 4.05 (dd, *J* = 11.6, 4.2 Hz, 1H, C*H*C(O)), 4.14 (d, *J* = 17.7 Hz, 1H, CH_a_*H_b_*C(O)N), 4.33 (d, *J* = 17.8 Hz, 1H, C*H_a_*H_b_C(O)N), 6.89 (s, 1H, C*H*Ph), 7.03 (t, *J* = 7.4 Hz, 1H, Ar-*H*), 7.12 (t, *J* = 7.6 Hz, 1H, Ar-*H*), 7.18 (d, *J* = 8.3 Hz, 2H, Ar-*H*), 7.35–7.30 (m, 1H, Ar-*H*), 7.53 (d, *J* = 7.7 Hz, 1H, Ar-*H*), 7.61–7.56 (m, 2H, Ar-*H*), 11.05 (s, 1H, NH). ESI-MS: m/z 456 (M^+^ + 2), m/z 454 (M^+^).

*(6S,12R)-2-(2-Amino-ethyl)-6-(4-bromo-phenyl)-2,3,6,7,12,12a-hexahydro-pyrazino[1’,2’:1,6]pyrido[3,4-b]indole-1,4-dione* (**7f**). Yellow solid. Yield: 41.7%. mp: 206–209 °C. TLC, R_f_: 0.49, (CH_2_Cl_2_/CH_3_OH/TEA, 95:4:1). IR: 3733.97, 3662.21 (NH_2_), 2968 (NH), 1657.4, 1650.1 (CO). ^1^H-NMR (Acetone-*d*_6_): δ 2.97 (ddd, *J* = 15.4, 11.9, 1.5 Hz, 1H, CH_a_*H_b_*), 3.37 (dd, *J* = 15.3, 4.3 Hz, 1H, C*H_a_*H_b_), 3.43 (dd, *J* = 14.6, 8.4 Hz, 2H, C*H_2_*NH_2_), 3.57–3.51 (m, 1H, NCH_a_*H_b_*CH_2_), 3.67–3.61 (m, 1H, NC*H_a_*H_b_CH_2_), 4.17 (dd, *J* = 11.9, 4.2 Hz, 1H, CHC(O)), 4.23 (d, *J* = 17.8 Hz, 1H, CH_a_*H_b_*C(O)N), 4.41 (dd, *J* = 17.8, 1.0 Hz, 1H, C*H_a_*H_b_C(O)N), 7.02 (s, 1H, C*H*Ph), 7.08 (ddd, *J* = 8.0, 7.1, 1.0 Hz, 1H, Ar-*H*), 7.15 (ddd, *J* = 8.2, 7.1, 1.2 Hz, 1H, Ar-*H*), 7.26–7.22 (m, 2H, Ar-*H*), 7.40–7.37 (m, 1H, Ar-*H*), 7.55–7.53 (m, 2H, Ar-*H*), 7.57 (dd, *J* = 7.8, 1.0 Hz, 1H, Ar-*H*). ESI-MS: m/z 455 (M^+^ + 2), m/z 453 (M^+^).

*(6S,12R)-6-(4-Bromo-phenyl)-2-(4-hydroxy-butyl)-2,3,6,7,12,12a-hexahydropyrazino[1’,2’:1,6]pyrido[3,4-b]indole-1,4-dione* (**7g**). White solid. Yield: 43%. mp: 207–209 °C. TLC, R_f_: 0.32, (CH_2_Cl_2_/CH_3_OH, 97:3). IR: 3100.62 (OH), 2920(NH), 1670.8, 1658.29 (CO). ^1^H-NMR (DMSO-*d*_6_): δ 1.45–1.33 (m, 2H, CH_2_C*H_2_*), 1.60–1.48 (m, 2H, C*H_2_*CH_2_), 2.99 (dd, *J* = 15.5, 12.5 Hz, 1H, CH_a_*H_b_*), 3.22–3.11 (m, 1H, NCH_a_*H_b_*CH_2_), 3.40 (t, *J* = 6.0 Hz, 2H, C*H_2_*OH), 3.55–3.44 (m, 1H, NC*H_a_*H_b_CH_2_), 4.06–3.97 (m, 2H, CH_a_*H_b_*C(O)N, CHC(O)), 4.30 (d, *J* = 17.6 Hz, 1H, C*H_a_*H_b_C(O)N), 6.88 (s, 1H, C*H*Ph), 7.03 (t, *J* = 7.4 Hz, 1H, Ar-*H*), 7.19–7.09 (m, 3H, Ar-*H*), 7.33 (d, *J* = 8.1 Hz, 1H, Ar-*H*), 7.60–7.51 (m, 3H, Ar-*H*), 11.06 (s, 1H, NH). ESI-MS: m/z 484 (M^+^ + 2), m/z 482 (M^+^).

*(6S,12R)-2-(4-Amino-butyl)-6-(4-bromo-phenyl)-2,3,6,7,12,12a-hexahydro-pyrazino[1’,2’:1,6]pyrido[3,4-b]indole-1,4-dione* (**7h**). White solid. Yield: 41%. mp: 210–212 °C. TLC, R_f_: 0.37, (CH_2_Cl_2_/CH_3_OH/TEA, 93:5:2). IR: 3666.41, 3542.31 (NH_2_), 2930.4 (NH), 1675.2, 1665.8 (CO). ^1^H-NMR (Acetone-*d*_6_): δ 1.60–1.54 (m, 2H, CH_2_C*H_2_*), 1.70–1.63 (m, 2H, C*H_2_*CH_2_), 2.98 (ddd, *J* = 15.4, 11.9, 1.5 Hz, 1H, CH_a_*H_b_*), 3.20 (t, *J* = 6.7 Hz, 2H, C*H_2_*NH_2_), 3.30 (dt, *J* = 13.7, 7.0 Hz, 1H, NCH_a_*H_b_*CH_2_), 3.37 (dd, *J* = 15.4, 4.3 Hz, 1H, C*H_a_*H_b_), 3.62–3.57 (m, 1H, NC*H_a_*H_b_CH_2_), 4.04 (d, *J* = 17.6 Hz, 1H, CH_a_*H_b_*C(O)N), 4.16 (dd, *J* = 11.8, 4.2 Hz, 1H, C*H*C(O)), 4.30 (dd, *J* = 17.6, 1.0 Hz, 1H, C*H_a_*H_b_C(O)N), 7.04 (s, 1H, C*H*Ph), 7.07 (ddd, *J* = 8.0, 7.1, 1.0 Hz, 1H, Ar-*H*), 7.16–7.12 (m, 1H, Ar-*H*), 7.25–7.22 (m, 2H, Ar-*H*), 7.40 (dt, *J* = 8.1, 0.9 Hz, 1H, Ar-*H*), 7.53–7.51 (m, 2H, Ar-*H*), 7.57–7.54 (m, 1H, Ar-*H*), 10.5 (s,1H, NH). ^13^C-NMR (Acetone-*d*_6_): 165.55, 163.4 (NCO), 139.77, 137.68, 132.49, 131.46, 130.73, 127.33, 122.87, 122.64, 120.13, 119.02, 112.17, 109.39, 53.65 (C6), 51.89 (C12), 49.89 (NC(O)*C*H_a_H_b_), 46.84 (NCH_a_H_b_), 46.13 (CH_2_NH_2_), 28.72 (*C*H_2_CH_2_), 27.86 (C*C*H_a_H_b_), 25.12 (CH_2_*C*H_2_).ESI-MS: m/z 483(M^+^ + 2), m/z 481 (M^+^).

*(6S,12S)-6-(4-Bromo-phenyl)-2-(2-hydroxy-ethyl)-2,3,6,7,12,12a-hexahydro-pyrazino[1’,2’:1,6]pyrido[3,4-b]indole-1,4-dione* (**7i**). Orange solid. Yield: 6%. mp: 233–236 °C. TLC, R_f_: 0.38, (CH_2_Cl_2_/CH_3_OH, 97:3). IR: 3056.91 (OH), 2920.61 (NH), 1656.8, 1650.29 (CO). ^1^H-NMR (Acetone-*d*_6_): δ 3.13 (ddd, *J* = 16.0, 11.6, 1.3 Hz, 1H, CH_a_*H_b_*), 3.49–3.45 (m, 1H, NCH_a_*H_b_*CH_2_), 3.62–3.59 (m, 1H, NC*H_a_*H_b_CH_2_), 3.65 (dd, *J* = 15.5, 4.3 Hz, 1H, C*H_a_*H_b_), 3.78–3.74 (m, 2H, *CH_2_*OH), 4.08 (d, *J* = 17.2 Hz, 1H, CH_a_*H_b_*C(O)N), 4.34 (dd, *J* = 17.2, 1.4 Hz, 1H, C*H_a_*H_b_C(O)N), 4.47 (dd, *J* = 11.5, 5.7 Hz, 1H, CHC(O)), 6.30 (s, 1H, C*H*Ph), 7.07–7.03 (m, 1H, Ar-*H*), 7.11–7.07 (m, 1H, Ar-*H*), 7.31–7.29 (m, 1H, Ar-*H*), 7.35–7.32 (m, 2H, Ar-*H*), 7.42–7.39 (m, 2H, Ar-*H*), 7.59 (dd, *J* = 4.5, 3.9 Hz, 1H, Ar-*H*), 10.18 (s,1H, NH). ESI-MS: m/z 456 (M^+^ + 2), m/z 454 (M^+^).

*(6S,12S)-2-(2-Amino-ethyl)-6-(4-bromo-phenyl)-2,3,6,7,12,12a-hexahydro-pyrazino[1’,2’:1,6]pyrido[3,4-b]indole-1,4-dione* (**7j**). White solid. Yield: 20%. mp: 232–236 °C. TLC, R_f_: 0.49, (CH_2_Cl_2_/CH_3_OH/TEA, 95:4:1). IR: 3733.97, 3662.21 (NH_2_), 2968 (NH), 1660.3, 1653.2 (CO). ^1^H-NMR (Acetone-*d*_6_): δ 2.95 (ddd, *J* = 15.4, 11.9, 1.5 Hz, 1H, CH_a_*H_b_*). 3.36 (dd, *J* = 15.4, 4.2 Hz, 1H, C*H_a_*H_b_), 3.45–3.39 (m, 2H, C*H_2_*NH_2_), 3.55–3.49 (m, 1H, NCH_a_*H_b_*CH_2_), 3.64–3.59 (m, 1H, NC*H_a_*H_b_CH_2_), 4.15 (dd, *J* = 11.8, 4.2 Hz, 1H, CHC(O)), 4.22 (d, *J* = 18.2 Hz, 1H, CH_a_*H_b_*C(O)N), 4.40 (dd, *J* = 17.8, 1.0 Hz, 1H, C*H_a_*H_b_C(O)N), 7.01 (s, 1H, C*H*Ph), 7.07 (ddd, *J* = 8.0, 7.1, 1.0 Hz, 1H, Ar-*H*), 7.14 (ddd, *J* = 8.2, 7.1, 1.2 Hz, 1H, Ar-*H*), 7.24–7.21 (m, 2H, Ar-*H*), 7.37 (dt, *J* = 8.1, 0.9 Hz, 1H, Ar-*H*), 7.54–7.51 (m, 2H, Ar-*H*), 7.57–7.54 (m, 1H, Ar-*H*), 10.2 (s,1H, NH). ESI-MS: m/z 455 (M^+^ + 2), m/z 453 (M^+^).

*(6S,12S)-6-(4-Bromo-phenyl)-2-(4-hydroxy-butyl)-2,3,6,7,12,12a-hexahydropyrazino[1’,2’:1,6]pyrido[3,4-b]indole-1,4-dione* (**7k**). Brown solid. Yield: 51%. mp: 234–236 °C. TLC, R_f_: 0.32, (CH_2_Cl_2_/CH_3_OH, 97:3). IR: 3100.62 (OH), 2920 (NH), 1670.8, 1658.29 (CO). ^1^H-NMR (Acetone-*d*_6_): δ 1.56–1.49 (m, 2H, CH_2_C*H_2_*), 1.71–1.64 (m, 2H, C*H_2_*CH_2_), 3.13 (ddd, *J* = 16.0, 11.6, 1.2 Hz, 1H, CH_a_*H_b_*), 3.46–3.39 (m, 1H, NCH_a_*H_b_*CH_2_), 3.60–3.54 (m, 3H, NC*H_a_*H_b_CH_2_ , C*H_2_*OH), 3.65 (dd, *J* = 16.1, 4.9 Hz, 1H, C*H_a_*H_b_), 3.93 (d, *J* = 17.1 Hz, 1H, CH_a_*H_b_*C(O)N), 4.24 (dd, *J* = 17.1, 1.3 Hz, 1H, C*H_a_*H_b_C(O)N), 4.46 (dd, *J* = 11.6, 3.7 Hz, 1H, CHC(O)), 6.30 (s, 1H, C*H*Ph), 7.07–7.03 (m, 1H, Ar-H), 7.10–7.07 (m, 1H, Ar-H), 7.31–7.29 (m, 1H, Ar-H), 7.35–7.32 (m, 2H, Ar-H), 7.43–7.40 (m, 2H, Ar-H), 7.60 (d, *J* = 7.3 Hz, 1H, Ar-H), 10.2 (s,1H, NH). ESI-MS: m/z 484 (M^+^ + 2), m/z 482 (M^+^).

*(6S,12S)-2-(4-Amino-butyl)-6-(4-bromo-phenyl)-2,3,6,7,12,12a-hexahydro-pyrazino[1’,2’:1,6]pyrido[3,4-b]indole-1,4-dione* (**7l**). Yellow solid. Yield: 34%. mp: 236–238 °C. TLC, R_f_: 0.37, (CH_2_Cl_2_/CH_3_OH/TEA, 93:5:2). IR: 3666.41, 3542.31 (NH_2_), 2930.4 (NH), 1680.2, 1669.6 (CO). ^1^H-NMR (Acetone-*d*_6_): δ 1.62–1.54 (m, 2H, CH_2_C*H_2_*), 1.69–1.63 (m, 2H, C*H_2_*CH_2_), 3.11 (ddd, *J* = 15.9, 11.6, 1.2 Hz, 1H, CH_a_*H_b_*), 3.21 (t, *J* = 6.7 Hz, 1H, C*H_2_*NH_2_), 3.44 (dt, *J* = 13.7, 7.0 Hz, 1H, NCH_a_*H_b_*CH_2_), 3.70–3.60 (m, 2H, NC*H_a_*H_b_CH_2_, C*H_a_*H_b_), 3.92 (d, *J* = 17.1 Hz, 1H, CH_a_*H_b_*C(O)N), 4.24 (dd, *J* = 17.0, 1.3 Hz, 1H, C*H_a_*H_b_C(O)N), 4.44 (dd, *J* = 11.5, 4.6 Hz, 1H, CHC(O)), 6.34 (s, 1H, C*H*Ph), 7.06–7.02 (m, 1H, Ar-*H*), 7.10–7.06 (m, 1H, Ar-*H*), 7.33–7.31 (m, 1H, Ar-*H*), 7.37–7.34 (m, 2H, Ar-*H*), 7.41–7.37 (m, 2H, Ar-*H*), 7.59–7.57 (m, 1H, Ar-*H*). ESI-MS: m/z 483 (M^+^ + 2), m/z 481 (M^+^).

*(6R,12S)-6-(4-Bromo-phenyl)-2-(2-hydroxy-ethyl)-2,3,6,7,12,12a-hexahydro-pyrazino[1’,2’:1,6]pyrido[3,4-b]indole-1,4-dione* (**7m**). Yellow solid. Yield: 45%. mp: 206–208 °C. TLC, R_f_: 0.38, (CH_2_Cl_2_/CH_3_OH, 97:3). IR: 3056.91 (OH), 2920.61 (NH), 1656.8, 1650.29 (CO). ^1^H-NMR (DMSO-*d*_6_): δ 3.00 (dd, *J* = 15.1, 12.1 Hz, 1H, CH_a_*H_b_*), 3.38 (dd, *J* = 8.9, 5.5 Hz, 2H, C*H_2_*OH), 3.55 (t, *J* = 5.4 Hz, 2H, NC*H_a_H_b_*CH_2_), 4.05 (dd, *J* = 11.7, 4.1 Hz, 1H, CHC(O)), 4.14 (d, *J* = 17.8 Hz, 1H, CH_a_*H_b_*C(O)N), 4.33 (d, *J* = 17.7 Hz, 1H, C*H_a_*H_b_C(O)N), 6.89 (s,1H, C*H*Ph), 7.03 (t, *J* = 7.4 Hz, 1H, Ar-*H*), 7.12 (t, *J* = 7.5 Hz, 1H, Ar-*H*), 7.18 (d, *J* = 8.5 Hz, 2H, Ar-*H*), 7.33 (d, *J* = 8.0 Hz, 1H, Ar-*H*), 7.58 (d, *J* = 8.3 Hz, 2H, Ar-*H*), 7.53 (d, *J* = 7.7 Hz, 1H, Ar-*H*), 11.05 (s, 1H, NH). ESI-MS: m/z 456 (M^+^ + 2), m/z 454 (M^+^).

*(6R,12S)-2-(2-Amino-ethyl)-6-(4-bromo-phenyl)-2,3,6,7,12,12a-hexahydro-pyrazino[1’,2’:1,6]pyrido[3,4-b]indole-1,4-dione* (**7n**). Yellow solid. Yield: 34%. mp: 206–210 °C. TLC, R_f_: 0.49, (CH_2_Cl_2_/CH_3_OH/TEA, 95:4:1). IR: 3733.97, 3662.21 (NH_2_), 2968 (NH), 1657.8, 1650 (CO). ^1^H-NMR (DMSO-*d*_6_): δ 3.08–2.99 (m, 1H,CH_a_*H_b_*), 2.72 (t, *J* = 6.4 Hz, 2H, NC*H_a_H_b_*CH_2_), 3.41 (dd, *J* = 13.2, 6.7 Hz, 2H, C*H_2_*NH_2_), 4.14–3.99 (m, 2H, CH_a_*H_b_*C(O)N, C*H*C(O)), 4.34 (d, *J* = 17.7 Hz, 1H, C*H_a_*H_b_C(O)N), 6.89 (s, 1H, C*H*Ph), 7.03 (t, *J* = 7.4 Hz, 1H, Ar-*H*), 7.12 (t, *J* = 7.6 Hz, 1H, Ar-*H*), 7.18 (d, *J* = 8.1 Hz, 2H, Ar-*H*), 7.33 (d, *J* = 8.1 Hz, 1H, Ar-*H*), 7.53 (d, *J* = 7.9 Hz, 1H, Ar-*H*), 7.58 (d, *J* = 7.7 Hz, 2H, Ar-*H*), 11.06 (s, 1H, NH). ESI-MS: m/z 455 (M^+^ + 2), m/z 453 (M^+^).

*(6R,12S)-6-(4-Bromo-phenyl)-2-(4-hydroxy-butyl)-2,3,6,7,12,12a-hexahydropyrazino[1’,2’:1,6]pyrido[3,4-b]indole-1,4-dione* (**7o**). Yellow solid. Yield: 30%. mp: 205–208 °C. TLC, R_f_: 0.32, (CH_2_Cl_2_/CH_3_OH, 97:3). IR: 3100.62 (OH), 2920(NH), 1671.8, 1660.9 (CO). ^1^H-NMR (DMSO-*d*_6_): δ 1.44–1.33 (m, 2H, C*H_2_*CH_2_), 1.60–1.49 (m, 2H, CH_2_C*H_2_*), 2.99 (dd, *J* = 14.9, 12.3 Hz, 1H, CH_a_*H_b_*), 3.15 (dd, *J* = 13.3, 7.0 Hz, 1H, NCH_a_*H_b_*CH_2_), 3.40 (t, *J* = 6.0 Hz, 2H, C*H_2_*OH), 3.48 (dd, *J* = 13.8, 7.1 Hz, 1H, NC*H_a_*H_b_CH_2_), 4.06–3.98 (m, 2H, CH_a_*H_b_*C(O)N, C*H*C(O)), 4.30 (d, *J* = 17.8 Hz, 1H, C*H_a_*H_b_C(O)N), 6.88 (s, 1H, C*H*Ph), 7.03 (t, *J* = 7.4 Hz, 1H, Ar-*H*), 7.11 (d, *J* = 8.0 Hz, 1H, Ar-*H*), 7.17 (d, *J* = 8.4 Hz, 2H, Ar-*H*), 7.33 (d, *J* = 8.1 Hz, 1H, Ar-*H*), 7.53 (d, *J* = 8.0 Hz, 1H, Ar-*H*), 7.58 (d, *J* = 8.2 Hz, 2H, Ar-*H*), 11.06 (s, 1H, NH). ESI-MS: m/z 484 (M^+^ + 2), m/z 482 (M^+^).

*(6R,12S)-2-(4-Amino-butyl)-6-(4-bromo-phenyl)-2,3,6,7,12,12a-hexahydro-pyrazino[1’,2’:1,6]pyrido[3,4-b]indole-1,4-dione* (**7p**). Yellow solid. Yield: 36%. mp: 210–212 °C. TLC, R_f_: 0.37, (CH_2_Cl_2_/CH_3_OH/TEA, 93:5:2). IR: 3666.41, 3542.31 (NH_2_), 2930.4 (NH), 1677.5, 1668.9 (CO). ^1^H-NMR (DMSO-*d*_6_): δ 1.37–1.26 (m, 2H, CH_2_C*H_2_*), 1.58–1.49 (m, 2H, C*H_2_*CH_2_), 2.54 (t, *J* = 6.9 Hz, 2H, C*H_2_*NH_2_), 2.99 (dd, *J* = 14.5, 12.2 Hz, 1H, CH_a_*H_b_*), 3.28 (dd, *J* = 15.4, 4.2 Hz, 1H, C*H_a_*H_b_), 3.52–3.41 (m, 1H, NC*H_a_*H_b_CH_2_), 4.08–3.99 (m, 2H, CH_a_*H_b_*C(O)N, C*H*C(O)), 4.29 (d, *J* = 17.3 Hz, 1H, C*H_a_*H_b_C(O)N), 6.88 (s, 1H, C*H*Ph), 7.06–7.00 (m, 1H, Ar-*H*), 7.15–7.09 (m, 1H, Ar-*H*), 7.17 (d, *J* = 8.4 Hz, 2H, Ar-*H*), 7.33 (d, *J* = 8.0 Hz, 1H, Ar-*H*), 7.53 (d, *J* = 7.6 Hz, 1H, Ar-*H*), 7.60–7.56 (m, 2H, Ar-*H*), 11.07 (s, 1H, NH). ESI-MS: m/z 483 (M^+^ + 2), m/z 481 (M^+^).

### 2.2. In-Vitro PDE5 Inhibitory Activity

Phosphodiesterase Assay: 5 U/mL of purified PDE5 (BPS Biosciences, San Diego, CA, USA) was added to the wells of black 96-well non-binding plates. Immediately, the protein was treated with the compound or vehicle control and 50 nM TAMRA-cGMP and 50 nM Fluourescein-cAMP (Molecular Devices, Sunnyvale, CA, USA) were added to each assay well. The plates were incubated for 1.5 h at 30 °C. After incubation, the IMAP FP Phosphodiesterase Evaluation Assay (Molecular Devices, Sunnyvale, CA, USA) binding reagent was added to each well and the plates were incubated for an additional 30 min at 30 °C. FP was measured according to manufacturer’s specifications using a Biotech Synergy 4 plate reader. PDE activity was expressed by an IC_50_ value (50% inhibitory concentration). Data analysis was done through dose-response analysis, which was repeated at least twice to confirm the reproducibility of the IC_50_ values.

### 2.3. Molecular Modeling

The docking experiment was implemented to dock **6c**, **6h**, & tadalafil into the active site of PDE5 with the program MOE version 2009.10. **6c**, **6h**, and tadalafil were constructed into the MOE window, then a conformational search was done for each of them using a systematic method, and the databases were saved. The Protein Data Bank (PDB) crystal structure of PDE5 co-crystallized with sildenafil (2H42) was imported into MOE. The structure was protonated and the binding pocket was selected and extended 4.5 A° around the pocket, the sildenafil molecule was then removed and tadalafil was loaded into MOE, where docking into the pocket was launched. The poses of the ligand conformation were generated using the alpha triangle, and the scoring function was alpha HB, increasing the hydrogen bond weight from 4 to 7. Each of the compounds **6c** and **6h** were docked using the same settings mentioned earlier, and they were superimposed on the best binding pose for tadalafil in order to compare the interactions between tadalafil and each compound. To ensure more accurate docking approaches, sildenafil was redocked to the binding pocket under the same MOE settings used for tadalafil, compound **6c** and **6h**.

## 3. Results and Discussion

### 3.1. Chemistry

**Scheme 1:**
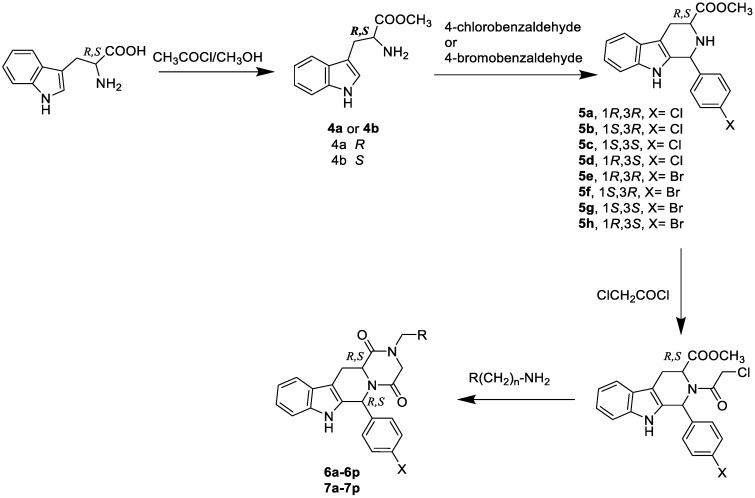
General scheme for the synthesis of novel PDE5 inhibitors.

The general synthesis of the tetrahydro-beta-carboline (THβC)-piperazinedione derivatives is illustrated in Scheme 1 and Table 1. d or l-tryptophan was esterified using methanol and acetyl chloride as a catalyst to produce the d or l-tryptophan methyl ester. Esters were reacted with *p*-chloro or *p*-bromo benzaldehyde in the presence of trifluoroacetic acid in a non-stereospecific reaction to produce the corresponding *cis* and *trans* THβCs which were separated using flash chromatography [16,17]. The pure *cis* and *trans* diastereomers were reacted with chloroacetylchloride to produce the corresponding chloroethanone derivatives. Finally, the chloroethanone intermediates were reacted with ethanolamine, ethylene diamine, 4-amino-1-butanol, and 1,4-diaminobutane producing the final THβC-piperazinedione derivatives. The structural elucidation of all synthesized compounds was done via different spectroscopic analytical techniques, such as Fourier transform infrared spectroscopy (FT-IR), Electrospray Ionisation (ESI) Mass Spectrometry, Nuclear Magnetic Resonance ^13^C-NMR and ^1^H-NMR spectroscopy. The IR spectra for all of the final compounds showed an absorption band at 2800–2950 cm^−1^ corresponding to the –NH bond stretching, it also showed two sharp bands at 1650–1670 cm^−1^ corresponding to the carbonyl (–C=O) stretching of the piperazinedione ring. For the final compounds with a terminal hydroxyl group, an additional weak band was shown at 3000–3150 cm^−1^ corresponding to the OH bond stretching. While for the final compounds with a terminal amino group, a fork-shaped band appeared at 3300–3500 cm^−1^ and this band corresponded to the –NH_2_ bond stretching of the terminal amino group. Mass spectra showed correct molecular ion peaks. The isotopic pattern was used to confirm the presence of the chloro- and bromo- substituent. Compounds having chlorine showed two ion peaks (M^+^ and M^+^ + 2) with peak height ratios of 3:1, while compounds having bromine showed two ion peaks (M^+^ and M^+^ + 2) with peak height ratios of 1:1. All the compounds showed purity above 95% using Liquid Chromatography-Mass Spectrometry LC-MS. ^13^C-NMR spectroscopy was used to assign *cis* and *trans* THβC after the Pictet-Spengler reaction. The signals for C1 and C3 for the *cis* diastereomer appeared more downfield than the corresponding carbon atoms in the *trans* diastereomer [16]. The ^1^H-NMR spectra confirmed the success of the Pictet-Spengler reaction through the appearance of a singlet peak at δ 6.32–7.22 ppm corresponding to the proton at C6. The incorporation of the terminal ethyl side chain attached to the nitrogen of the piperazinedione was confirmed through the presence of two multiple peaks at δ 3.85–3.00 ppm, each corresponding to one proton on the carbon next to the nitrogen atom of the piperazinedione ring (N–C*H_2_*–), and a multiplet or a doublet of a doublet peak at δ 3.85–3.00 ppm, corresponding to two protons on the carbon adjacent to the terminal hydrophilic group (–C*H_2_*OH). The incorporation of the butyl side chain was confirmed through the presence of two additional multiplet peaks, each integrating two protons at δ 1.80–1.30 ppm.

### 3.2. In Vitro PDE5 Inhibitory Activity

All the synthesized final THβC compounds were tested for their ability to inhibit recombinant human PDE5. Most of the compounds were evaluated in two steps. The first step was the determination of the percentage inhibition at a screening dose of 10 μM performed in triplicate. For compounds displaying a percentage of inhibition >50%, the IC_50_ was then determined by testing a range of 10 concentrations with at least two replicates per concentration (Table 2).

Fifteen of the 32 active compounds showed PDE5 inhibitory activity with IC_50_ values between 0.1–4.7 µM. The active novel PDE5 inhibitors for both the *p*-chloro and the *p*-bromo series having a terminal hydrophilic group and an R configuration at C6, **6b**, **6c**, **6o**, **7a**, **7b**, **7c**, **7d**, **7m**, **7n**, **7o**, and **7p** were less active than the reported tadalafil analogs with the same absolute configuration and equal N-alkyl spacer, lacking the terminal hydrophilic group. These compounds were less active than tadalafil itself (IC_50_ = 2 nM) and were also less active than the corresponding tadalafil derivatives with the benzodioxol moiety at C6 and an equal N-alkyl spacer. The active novel PDE5 inhibitors for both the *p*-chloro and the *p*-bromo series having a terminal hydrophilic group but an *S* configuration at C6 were compounds (**6e**, **6h**, **6k**, and **7h**), which were more active than the corresponding tadalafil analogs, which lack the terminal hydrophilic group and have the same *N*-alkyl spacer with the same absolute configuration. These compounds were also more active than the tadalafil molecules having an S configuration at C6, where (IC_50_ > 10 μM) [16]. These results prove that the analogues’ hydrophilic substituents succeeded in picking up new interactions even when compounds had an inverted stereocenter and suffered from the loss of the commonly reported interactions of PDE5 inhibitors.

Upon comparison of the active novel PDE5 inhibitors, it was found that using a *p*-bromo phenyl substitution at C6 yielded more potent PDE5 inhibitors than using the *p*-chloro phenyl one.

By comparing the terminal amino and hydroxyl groups, it was found that the hydroxyl group yielded more potent PDE5 inhibitors. As for the spacer length between the nitrogen atom of the piperazinedione and the terminal hydrophilic group, using a four-carbon atom spacer yielded more potent compounds than when using a two-carbon atom spacer. This was obvious when observing the most active compound in the *p*-chloro series, **6c**, IC_50_ = 0.12 µM, and the most active compound in the *p*-bromo series, **7c**, IC_50_ = 0.1 µM, where both have an N-butyl hydroxyl terminal substitution. Finally regarding the stereochemistry, the most active compounds having an IC_50_ value of <1 µM, all had an *R* configuration at C6 regardless of the absolute configuration at C12a. The active compounds having an *S* configuration at C6, **6e**, **6h**, **6k**, and **7h**, have shown promising activity, as they were more active than the corresponding tadalafil analog congeners with terminal hydrophobic groups [16]. The observed results support our approach of imparting activity to the inactive *S* isomer by appropriate selection of the side chain on the terminal nitrogen.

### 3.3. Molecular Modeling Studies

**Figure 3:**
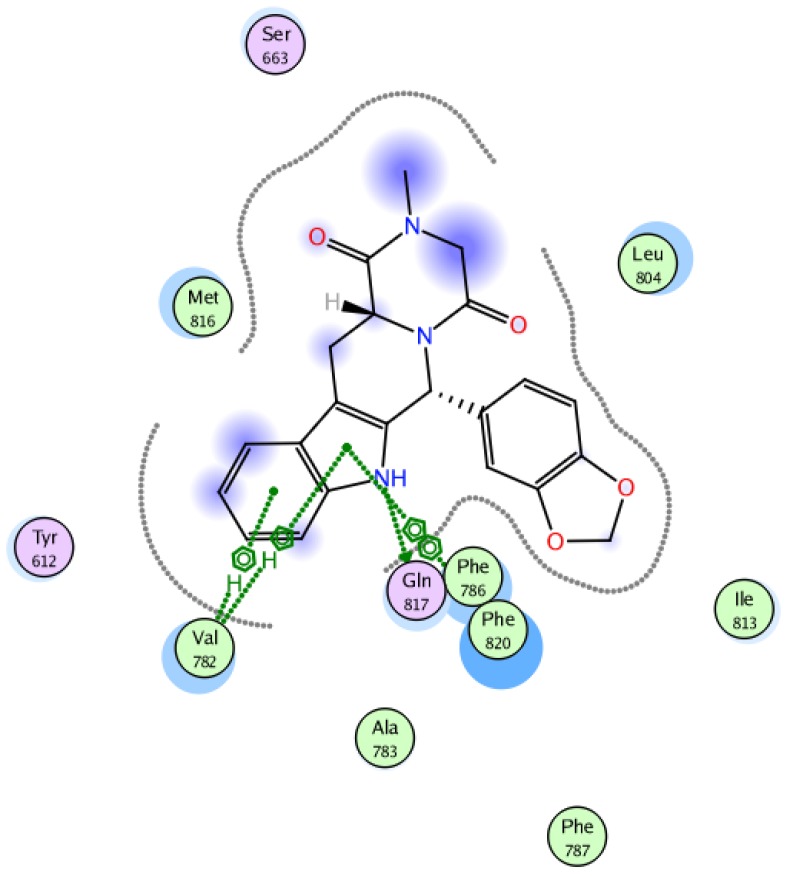
2D view showing the docking and tadalafil with the PDE5 active site. Purple rounds indicate residues with polar uncharged side chain; green rounds indicate residues with hydrophobic side chains

**Figure 4:**
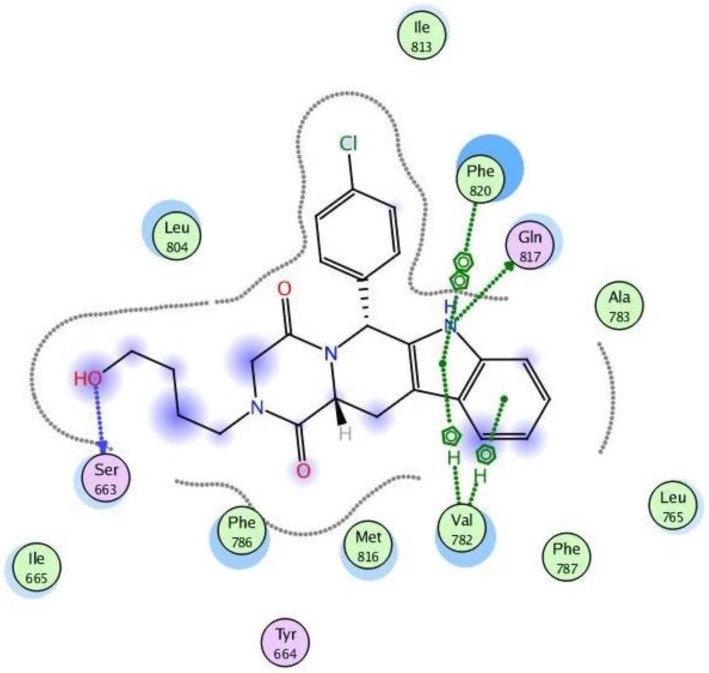
2D view showing the docking and interaction of compound **6c** with the PDE5 active site.

**Figure 5:**
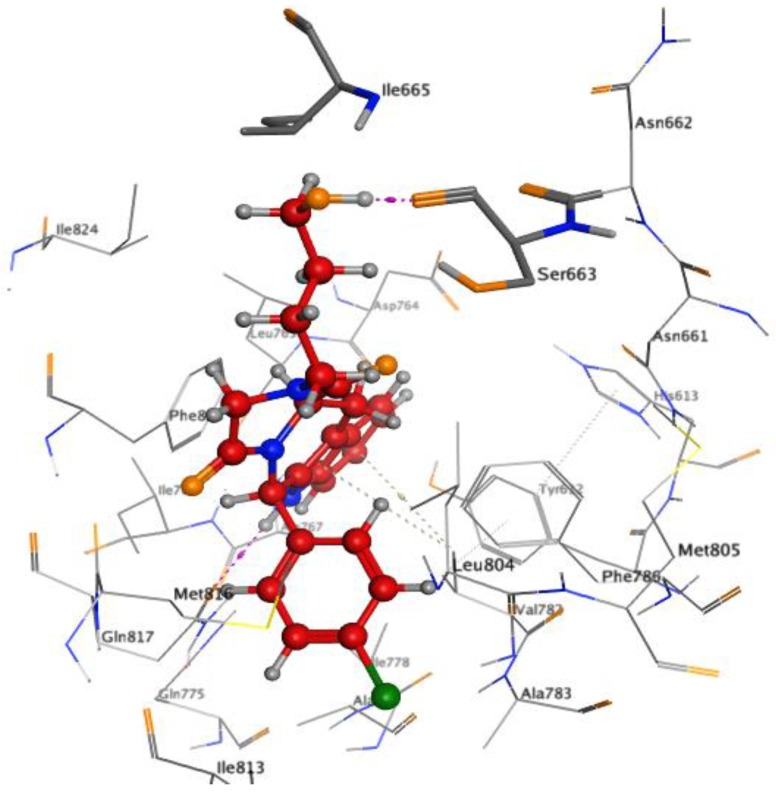
Docked pose of compound **6c** (red) in the cavity of 2H42, interaction purple, featuring a hydrogen bond with **Ser663**.

In order to validate our hypotheses, tadalafil and compound **6c**, the most active compounds in the *p*-chloro series, were docked in the binding pocket of PDE5 (PDB code 2H42), using the Molecular Operating Environment (MOE) software. A 2D view of the interaction between tadalafil and the PDE5 binding pocket (Figure 3) was compared to the interaction of compound **6c** with the residues lining the binding pocket of PDE5 (Figure 4). Both tadalafil and compound 6c formed essential interactions that include; a hydrogen bond with **Gln817**, π–π stacking, and hydrophobic interactions with **Phe820** and **Val782**. However, the hydrophilic group in compound **6c** picked up a hydrogen bond with **Ser663** instead of being oriented towards **Ile665** as shown in Figure 4 and Figure 5.

**Figure 6:**
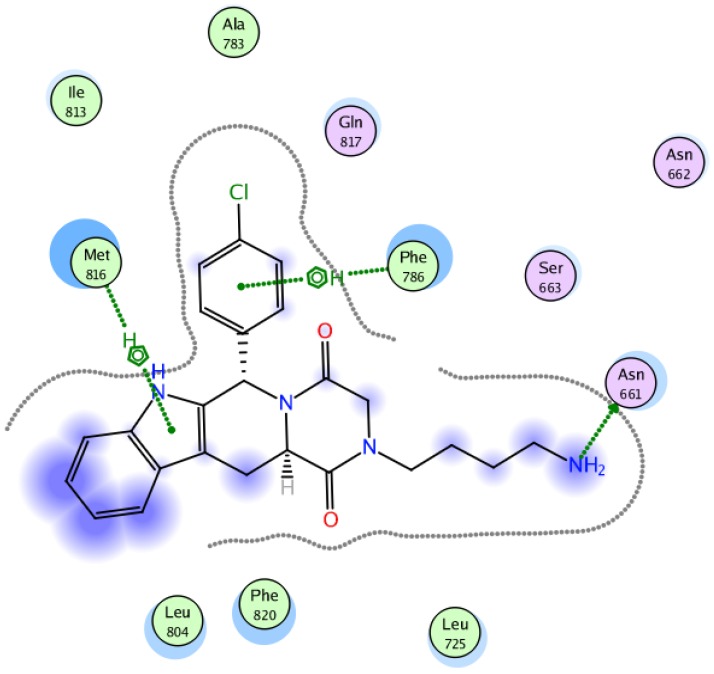
2D view showing the docking of compound **6h** with the PDE5 active site.

**Figure 7:**
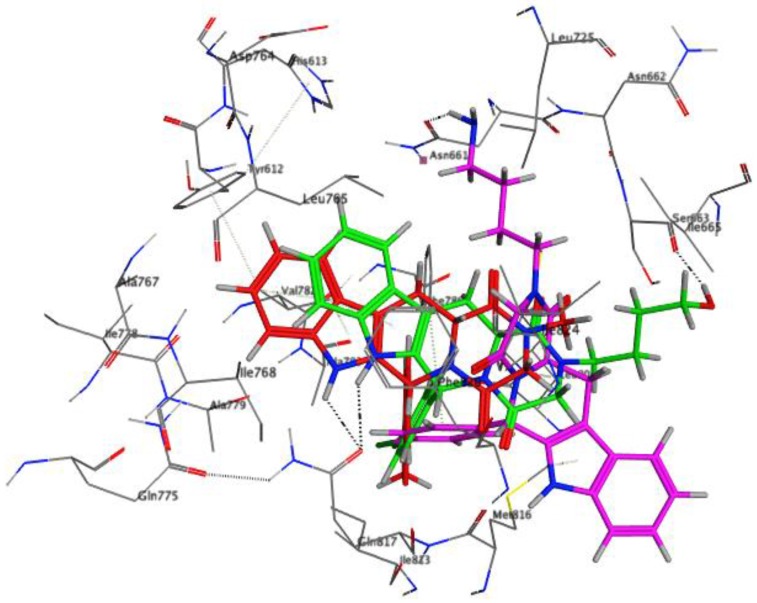
Docked pose of compound **6h** (red) in the cavity of 2H42, purple, featuring a hydrogen bond with **Asn661**.

**Figure 8:**
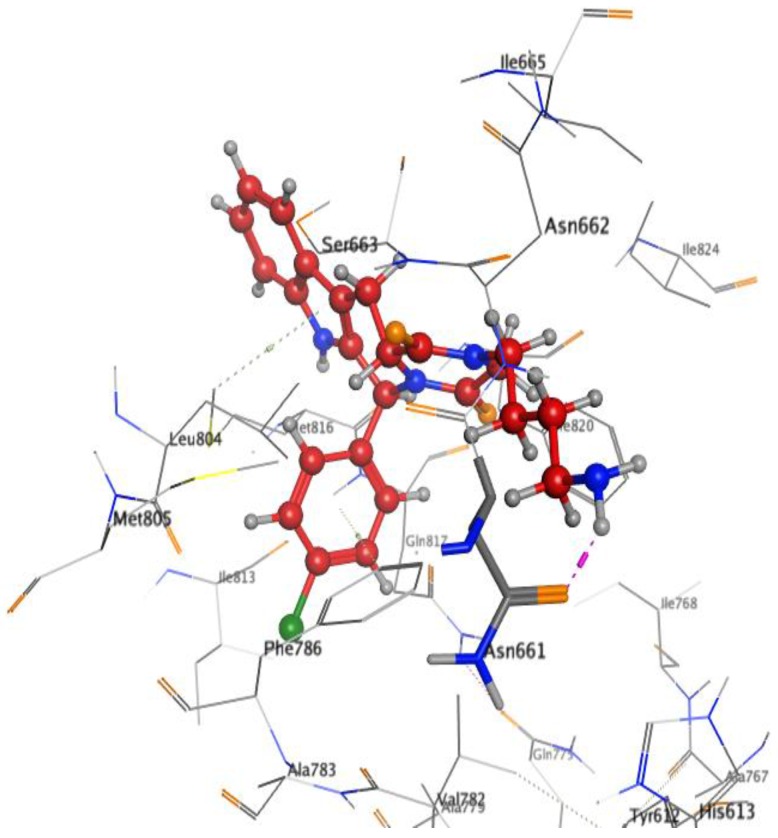
Docked pose of tadalafil (red), compound **6c** (green), compound **6h** (purple), in the cavity of 2H42.

This failure to exhibit a hydrophobic interaction with **Ile665** led to a 100-fold decrease in potency compared to tadalafil. A possible interpretation for such low potency may be attributed to the crucial role of **Ile665** in PDE5 inhibition. To gain an insight on the effect of the stereochemistry of C6 to inhibit PDE5, compound **6h**, one of the active compounds despite having an *S*-configuration at C6, was docked at the binding pocket of PDE5 (PDB code 2H42), using the MOE software. A 2D view of the interaction of compound **6h** with the PDE5 active site as shown in Figure 6 (IC_50_ = 3.1 µM) and despite losing all the essential interactions, succeeded in developing a new interaction with Asn661, one of the three hydrophilic residues with a polar uncharged side chain present beside the Ile665 residue. This new interaction might have compensated partially for the loss of the other essential interactions. Further confirmation is shown in the 3D docking picture of tadalafil and compound **6h** with the PDE5 active site in Figure 7 and Figure 8. This confirms the success of our structure-based approach in providing new interactions with hydrophilic residues in the side chain.

## 4. Conclusions

From these data, we conclude that the addition of a terminal hydrophilic group at the nitrogen of the piperazinedione improved the activity for the compounds having an *S* configuration at C6, previously reported as inactive congeners, where it interacts with one of the residues having a polar side chain (**Ser663**, **Asn662**, and **Asn661**). However, the addition of a terminal hydrophilic group at the nitrogen of the piperazinedione seemed to decrease the activity of the compounds having an *R* configuration at C6 with the N-substituent bearing an alkyl/aryl hydrophobic group. This decrease in activity can be explained by the fact that the terminal hydrophilic group interrupted/abolished the hydrophobic interaction with **Ile665**. These data prove the importance and the influence of the **Ile665** interaction on the PDE5 inhibitory activity and further validate our hypothesis. On the other hand, interactions with hydrophilic residues in the binding residue partially compensate for the activity loss.

## Figures and Tables

**Table 1:** Synthesized compounds.

Cpd#	Stereo-Chemistry	X	R	n	% Yield	Cpd#	Stereo-Chemistry	X	R	n	% Yield
**6a**	6*R*,12a*R*	Cl	OH	2	10	**7a**	6*R*,12a*R*	Br	OH	2	37
**6b**	6*R*,12a*R*	Cl	NH_2_	2	14	**7b**	6*R*,12a*R*	Br	NH_2_	2	13
**6c**	6*R*,12a*R*	Cl	OH	4	48	**7c**	6*R*,12a*R*	Br	OH	4	56
**6d**	6*R*,12a*R*	Cl	NH_2_	4	10	**7d**	6*R*,12a*R*	Br	NH_2_	4	38
**6e**	6*S*,12a*R*	Cl	OH	2	26	**7e**	6*S*,12a*R*	Br	OH	2	55
**6f**	6*S*,12a*R*	Cl	NH_2_	2	10	**7f**	6*S*,12a*R*	Br	NH_2_	2	42
**6g**	6*S*,12a*R*	Cl	OH	4	64	**7g**	6*S*,12a*R*	Br	OH	4	43
**6h**	6*S*,12a*R*	Cl	NH_2_	4	30	**7h**	6*S*,12a*R*	Br	NH_2_	4	41
**6i**	6*S*,12a*S*	Cl	OH	2	40	**7i**	6*S*,12a*S*	Br	OH	2	10
**6j**	6*S*,12a*S*	Cl	NH_2_	2	30	**7j**	6*S*,12a*S*	Br	NH_2_	2	20
**6k**	6*S*,12a*S*	Cl	OH	4	31	**7k**	6*S*,12a*S*	Br	OH	4	51
**6l**	6*S*,12a*S*	Cl	NH_2_	4	27	**7l**	6*S*,12a*S*	Br	NH_2_	4	34
**6m**	6*R*,12a*S*	Cl	OH	2	20	**7m**	6*R*,12a*S*	Br	OH	2	45
**6n**	6*R*,12a*S*	Cl	NH_2_	2	48	**7n**	6*R*,12a*S*	Br	NH_2_	2	34
**6o**	6*R*,12a*S*	Cl	OH	4	44	**7o**	6*R*,12a*S*	Br	OH	4	30
**6p**	6*R*,12a*S*	Cl	NH_2_	4	50	**7p**	6*R*,12a*S*	Br	NH_2_	4	36

**Table 2:** Inhibitory activity of the synthesized compounds on PDE5.

Cpd#	PDE5 Inhibition IC_50_ (µM)	Cpd#	PDE5 Inhibition IC_50_ (µM)
**6a**	>10	**7a**	0.84
**6b**	3.6	**7b**	2.4
**6c**	0.12	**7c**	0.1
**6d**	>10	**7d**	2.7
**6e**	4.7	**7e**	>10
**6f**	>10	**7f**	>10
**6g**	>10	**7g**	>10
**6h**	3.1	**7h**	3.2
**6i**	>10	**7i**	>10
**6j**	>10	**7j**	>10
**6k**	2.31	**7k**	>10
**6l**	>10	**7l**	>10
**6m**	>10	**7m**	0.6
**6n**	>10	**7n**	0.9
**6o**	2.31	**7o**	0.82
**6p**	>10	**7p**	3.5
